# Alternatively Spliced Homologous Exons Have Ancient Origins and Are Highly Expressed at the Protein Level

**DOI:** 10.1371/journal.pcbi.1004325

**Published:** 2015-06-10

**Authors:** Federico Abascal, Iakes Ezkurdia, Juan Rodriguez-Rivas, Jose Manuel Rodriguez, Angela del Pozo, Jesús Vázquez, Alfonso Valencia, Michael L. Tress

**Affiliations:** 1 Structural Biology and Bioinformatics Programme, Spanish National Cancer Research Centre (CNIO), Madrid, Spain; 2 Unidad de Proteómica, Centro Nacional de Investigaciones Cardiovasculares (CNIC), Madrid, Spain; 3 National Bioinformatics Institute (INB), Spanish National Cancer Research Centre (CNIO), Madrid, Spain; 4 Instituto de Genetica Medica y Molecular, Hospital Universitario La Paz, Madrid, Spain; 5 Laboratorio de Proteómica Cardiovascular, Centro Nacional de Investigaciones Cardiovasculares (CNIC) Madrid, Spain; Royal Institute of Technology, SWEDEN

## Abstract

Alternative splicing of messenger RNA can generate a wide variety of mature RNA transcripts, and these transcripts may produce protein isoforms with diverse cellular functions. While there is much supporting evidence for the expression of alternative transcripts, the same is not true for the alternatively spliced protein products. Large-scale mass spectroscopy experiments have identified evidence of alternative splicing at the protein level, but with conflicting results. Here we carried out a rigorous analysis of the peptide evidence from eight large-scale proteomics experiments to assess the scale of alternative splicing that is detectable by high-resolution mass spectroscopy. We find fewer splice events than would be expected: we identified peptides for almost 64% of human protein coding genes, but detected just 282 splice events. This data suggests that most genes have a single dominant isoform at the protein level. Many of the alternative isoforms that we could identify were only subtly different from the main splice isoform. Very few of the splice events identified at the protein level disrupted functional domains, in stark contrast to the two thirds of splice events annotated in the human genome that would lead to the loss or damage of functional domains. The most striking result was that more than 20% of the splice isoforms we identified were generated by substituting one homologous exon for another. This is significantly more than would be expected from the frequency of these events in the genome. These homologous exon substitution events were remarkably conserved—all the homologous exons we identified evolved over 460 million years ago—and eight of the fourteen tissue-specific splice isoforms we identified were generated from homologous exons. The combination of proteomics evidence, ancient origin and tissue-specific splicing indicates that isoforms generated from homologous exons may have important cellular roles.

## Author Summary

Alternative splicing is thought to be one means for generating the protein diversity necessary for the whole range of cellular functions. While the presence of alternatively spliced transcripts in the cell has been amply demonstrated, the same cannot be said for alternatively spliced proteins. The quest for alternative protein isoforms has focused primarily on the analysis of peptides from large-scale mass spectroscopy experiments, but evidence for alternative isoforms has been patchy and contradictory. A careful analysis of the peptide evidence is needed to fully understand the scale of alternative splicing detectable at the protein level. Here we analysed peptides from eight large-scale data sets, identifying just 282 splice events among 12,716 genes. This suggests that most genes have a single dominant isoform. Many of the alternative isoforms that we identified were only subtly different from the main splice variant, and one in five was generated by substitution of homologous exons by swapping one related exon for another. Remarkably, the alternative isoforms generated from homologous exons were highly conserved, first appearing 460 million years ago, and several appear to have tissue-specific roles in the brain and heart. Our results suggest that these particular isoforms are likely to have important cellular roles.

## Introduction

Studies have estimated that alternative splicing can produce differently spliced messenger RNA (mRNA) transcripts for practically all multi-exon human genes [1,2]. These mRNA variants have the potential to expand the cellular protein repertoire far beyond the one gene–one protein model that formed part of the central dogma for many years [3,4].

The number of alternatively spliced transcripts annotated in reference human gene sets has grown steadily in recent years and manual genome annotation projects such as GENCODE [5] are identifying ever more alternative variants. The current version of the GENCODE gene set annotates more than 93,000 protein-coding variants, a number that has increased by 10,000 since 2009.

Theoretically all these transcripts could be translated into functional protein isoforms and could greatly diversify the cellular functional repertoire. However, although we have a limited understanding of the function of a small number of these alternative isoforms, there is a general lack of knowledge about the functional roles of the vast majority of annotated splice isoforms in the cell. All we can say is that most of the annotated splice variants in the human genome will produce isoforms with substantially altered 3D structure and consequent drastic change of biological function, if translated to protein [6,7].

There is considerable supporting evidence for the generation of multiple alternative mRNA transcripts from the same gene. EST and cDNA sequence evidence [8], microarray data [9] and RNAseq data [10] strongly support alternative splicing at the mRNA transcript level.

In spite of the overwhelming evidence of alternative splicing at the transcript level, there is limited support for the translation of these alternative transcripts into protein isoforms. Individual experiments can provide evidence for the expression of isoforms for single genes [11]. At the genome level large-scale antibody tagging [10] holds promise for the detection of alternative isoforms, but the broad specificity of most antibodies makes their discrimination almost impossible at present. For antibody tagging to be of use in distinguishing alternative isoforms, they should be designed from the beginning with this purpose in mind, and each antibody should be only capable of detecting one splice event in one protein. Ribosome profiling experiments [12] have been used in recent years as a proxy for protein coding potential [13,14], but ribosome-profiling data should be used with caution [15] not least because ribosome-profiling methods require transcript reconstruction algorithms to predict splicing variants. The reliability of these transcript reconstruction algorithms has recently been thrown into doubt [16–18]. These factors may have lead research groups to reach two entirely different and opposing conclusions from the same ribosome profiling data [19,20].

High-throughput tandem mass spectrometry (MS)-based proteomics [21] is the main source of peptide evidence. Reliable proteomics data can confirm transcript coding potential even where there is little other supporting evidence. MS-based proteomics has become an increasingly important tool in genome annotation thanks to advances over the last two decades and a number of groups have demonstrated how proteomics data might be properly used to validate translation of protein coding genes [22–24]. On a larger scale, the Human Proteome Project [11] is attempting to identify at least one protein product for each human gene.

Several groups have now identified small numbers of alternative protein isoforms in species ranging from human [22] to mouse [23], *Drosophila* [25], *Arabidopsis* [26] and *Aspergillus flavus* [27]. Recently two large-scale analyses produced similar results. Our group [24] detected the expression of multiple splice isoforms for 150 of 7,597 human genes from an analysis of spectra from the GPM [28] and PeptideAtlas [29] databases, while Low et al. [30] identify 83 alternative splicing events and 13,088 genes in rat.

By way of contrast, a number of recent proteomics studies claim to have found substantially more cases of alternative splicing at the protein level. Menon *et al* [31] identified 420 alternative isoforms from 1,278 mouse genes, but at the time the mouse genome was not well annotated and it is not clear whether this study required peptides to identify both constitutive and alternative splice isoforms. Recently the numbers of identified splice isoforms have escalated substantially. In two papers published in the same issue of Nature, Kuster and co-workers [32] identified 1,279 alternative proteins for more than 18,097 human genes, while Pandey and colleagues found “isoform-specific peptides” for 2,861 protein isoforms from more than 17,294 genes [33]. As we have shown [34], the main problem with these studies is that they dramatically overestimate the number of reliable peptide identifications. At the extreme end of the scale Ly *et al* claim to have found evidence for 33,575 separate protein isoforms from just 12,000 human genes [35], suggesting that they identified more than 21,000 alternative isoforms, an order of magnitude greater than any previous study. Here the authors did not use discriminating peptides, but instead chose to infer the expression of different isoforms based on peptide abundances in an analogous way to the protocols used for transcript level estimation in RNAseq studies [16,17]. This form of identifying alternative protein isoforms is wholly inappropriate in proteomics studies because of the low peptide coverage typical of these experiments and because of the non-uniform distribution of the peptides detected.

Given the wide variety in the numbers of splice isoforms reported in what are essentially similar, large-scale proteomics experiments, we felt that it was important to carry out a rigorous study of alternative splicing at the protein level. To accomplish this we produced as reliable a set of peptides as possible from eight high-throughput MS analyses. These analyses were carried out on a wide range of cell types.

We identify alternative splice isoforms for 246 genes from the reliable peptide evidence from the eight data sets. We demonstrate that this is far below what would be expected if the main and alternative splice isoforms were produced in comparable quantities in the cell, suggesting that most genes have a single main protein isoform. We found that homologous exons substitutions, consecutive exons that are homologous and are spliced in a mutually exclusive manner, were highly enriched among the splicing events that we did identify and we show that remarkably few of the events we identified affected the composition of functional domains.

## Results

Peptides for the human dataset were collected from eight distinct large-scale proteomics analyses. Two came from proteomics databases, the high quality peptide identifications from the PeptideAtlas database [21] and NIST (http://peptide.nist.gov/), a compendium of peptides identified in multiple proteomics experiments by multiple search engines. The other six correspond to recently published large-scale experiments [32,33,36–39]. These eight datasets cover a large range of tissues and cell types; the peptides from the PeptideAtlas database cover 51 different tissue types and developmental stages. In total the eight datasets contained peptides from over 100 distinct tissues and cell lines. All references to the separate peptide data sets or experiments are by first author name in the text and further details for each individual set after filtering can be found in S1 Table.

The accuracy of peptide identification is a complex issue. Here we decided to work with a conservative set of high quality peptides. In order to generate as reliable a set of peptides as possible we filtered the peptides from each experiment by excluding all non-tryptic and semi-tryptic peptides and all peptides containing missed cleavages that were not supported by at least one of the fully cleaved tryptic peptides. Where possible we included only peptides that were identified by at least two search engines (see Materials and Methods section for details of the individual experiments). The full details of all the filters are explained in the Materials and Methods section.

After filtering we obtained a total of 277,244 unique, high-quality peptides. The number of peptide identifications from each analysis can be seen in S1 Table. We mapped the peptides from the eight analyses to the GENCODE 20 annotation of the human genome (GENCODE 20 corresponds to Ensembl 76 [40] and is the first annotation from build 38 of the human genome). We detected at least two peptides for 12,716 genes, a total of 63.9% of the annotated protein coding genes.

It should be noted that 2,807 of these peptides (1%) did not match GENCODE 20 genes. Almost 75% of this subpopulation of peptides were only detected in one of the eight analyses (compared to 46% of those peptides that do map to annotated coding genes), so a significant proportion are likely to be false positive matches. Peptides detected in two or more experiments have been sent to the GENCODE annotators for further study.

**Fig 1:**
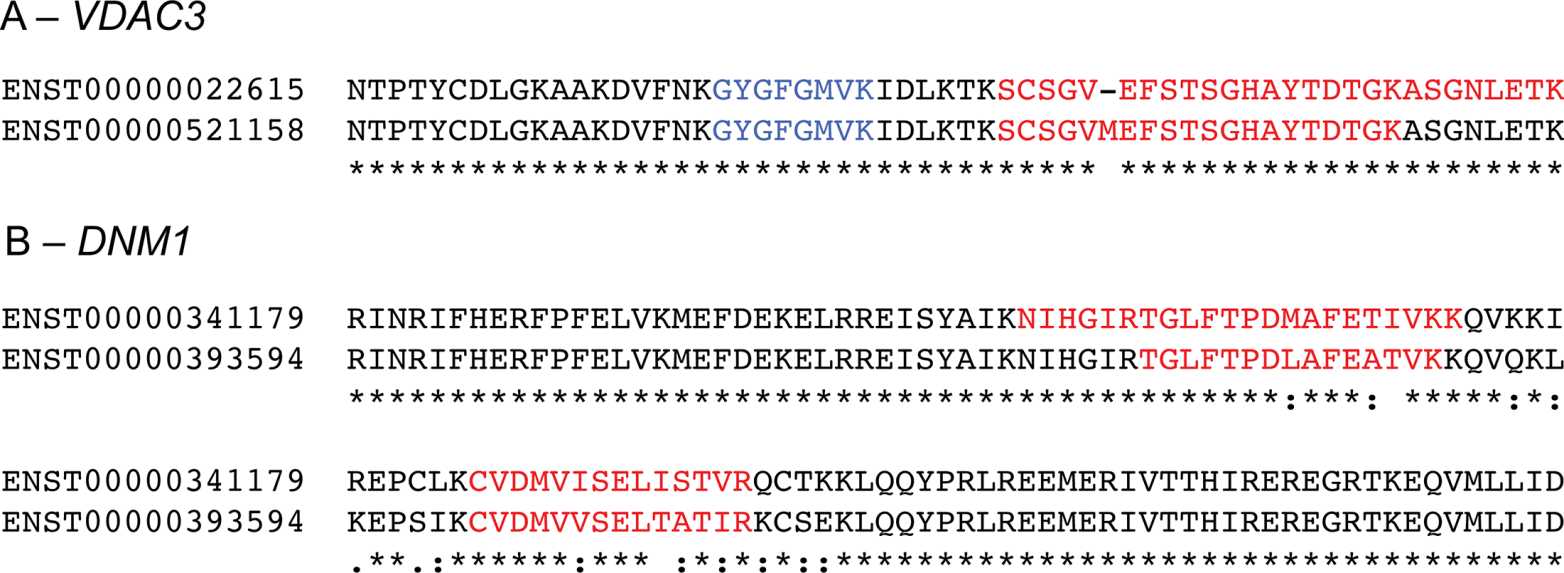
Splice event specific peptides for *VDAC3* and *DNM1*. A. Pairwise alignment between two variants from the *VDAC3* gene, ENST00000022615 and ENST00000521158. The peptides from the eight data sets are mapped onto the alignment, blue when they do not discriminate between the two splice isoforms, red when they do. The two *VDAC3* isoforms differ by a single inserted residue. Two peptides mapped uniquely to variant ENST00000022615; one was detected in all eight data sets, the other was a supported missed cleavage and found in three data sets. Variant ENST00000521158 was supported by a single peptide that was identified in four of the data sets. B. The pairwise alignment between two variants from the *DNM1* gene, ENST00000341179 and ENST00000393594, a case of mutually exclusive homologous splicing. The peptides identified in the eight data sets are mapped onto the alignment, two peptides supported each splice isoform.

We only considered those peptides that mapped unequivocally to a single gene (discriminating peptides) and only used peptides that were detected in two or more of the eight large-scale analyses in order to reduce the number of false positive identifications. In total 149,612 gene discriminating peptides were detected in multiple analyses; of these peptides 111,382 (74.3%) were able to discriminate between at least two isoforms. To detect peptide evidence of splice isoforms, we required peptides to map to regions that distinguished both sides of a splicing event, whether the splicing event was an indel (Fig 1A) or a substitution event (Fig 1B). To identify deletions we required the peptide to cross the exon boundary. For insertions or substitutions, peptides were permitted to map to any part of the insertion/substitution.

### Prevalence of different types of alternative splicing in proteomics experiments

Over the 8 experiments we identified splicing events from 246 genes. This is 60% more than our previous study, in which we reported peptide evidence for 150 genes [24]. We identified splicing events for 77 of these 150 genes in this analysis.

Twenty genes had evidence for more than one alternative splice isoform, and three genes (*PLEC*, *TPM1* and UGT1A) had evidence for five or more different splice isoforms. Here the UGT1A gene cluster is defined as a single gene, even though it is annotated as a cluster of nine independent genes in the GENCODE gene set. These “genes” differ in a set of mutually exclusive 5’ exons that are joined to a common set of 3’ exons by alternative splicing, so we have treated them, and four similar GENCODE 20 gene clusters, as splice variants of a single gene (see Materials and Methods). There was peptide evidence for 8 different protein isoforms from the UGT1A gene (S1 Fig).

To take into account multiple splice isoforms from the same gene, we carried out our analysis on alternative splicing events rather than genes. Alternative splicing events are those that differentiate the alternative isoform from the main isoform (the isoform for which we identify most discriminating peptides). Alternative splicing events identified in the analysis are referred to as identified splicing events (ISE). We found peptide evidence for 282 different ISEs from the 246 genes (S2 Table).

The ISE were classified by their effect on the protein since this is more relevant for a study at the protein level. Events were classified as (i) indels–insertion or deletion of more than 4 amino acid residues (S2 Fig); (ii) NAGNAG splicing [41], defined as the insertion or deletion of up to four amino acid residues (Fig 1); (iii) homologous substitutions (Figs 1 and S3): (iv) other C-terminal substitutions (S4 Fig); (v) other N-terminal substitutions (S5 Fig); (vi) internal non-homologous substitutions (S6 Fig); and (vii) generating two non-homologous proteins (S7 Fig).

**Fig 2:**
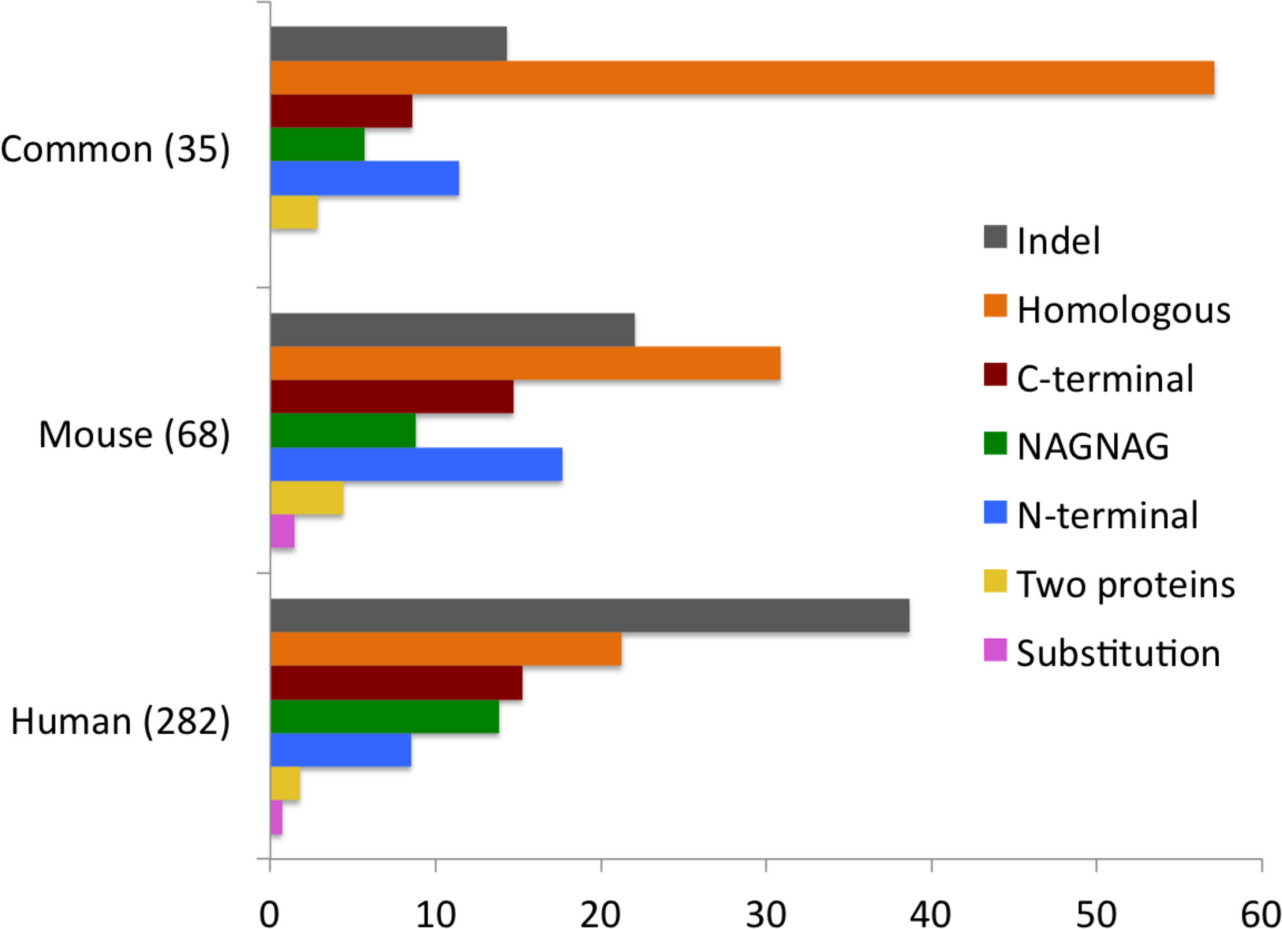
Splice events identified in the mouse and human experiments. The percentages of each type of splice event in the human analysis (8 protein data sets), the mouse analysis (three data sets) and among those splice events that were common to both analyses. We identified 282 and 68 events in human and mouse respectively and there were 35 splice events in common to both analyses.

The majority of the ISE were indels (109 ISE); this is to be expected since most annotated alternative splicing events are indels [42, 43]. The second most common alternative splicing events were homologous substitutions (60 ISE), followed by non-homologous C-terminal substitutions (43 ISE). There were also numerous alternative splice events involving GYNGYN donors or NAGNAG acceptors (39 ISE). These result in small insertions and are referred to in the paper as NAGNAG splicing events. There were fewer non-homologous N-terminal substitution events (24 ISE), while internal non-homologous substitutions (2 ISE) and events that generated distinct proteins (5 ISE) were less frequent (Fig 2).

### Alternative splice isoforms in mouse

We carried out a similar analysis with mouse. We would expect to identify fewer alternative splice variants because the mouse genome is annotated with fewer alternative isoforms (see the Materials and Methods section) and because we only interrogated three analyses, the equivalent peptides from the NIST and PeptideAtlas databases, and an in-house analysis of the mouse spectra in the PeptideAtlas and GPM databases.

As with the human analysis we required all peptides to have been identified in at least two analyses (a more stringent requirement because in this case there were only three proteomics analyses). We identified splice isoforms for 56 genes and 68 splicing events. We detected the identical splice event in the orthologous human gene for 35 of the 68 mouse ISEs. We also detected another 29 mouse events that were equivalent to human ISEs but that were only supported by peptides from a single analysis.

Twenty-one of the 68 mouse ISE (30.1%) we detected were generated from homologous exons and all but one of these 21 splicing events also had peptide evidence in the orthologous human genes. Hence, homologous substitutions are particularly highly represented among those splice events detected in both human and mouse (Fig 2), and make up almost 60% of the orthologous splicing events we detected in both human and mouse experiments.

### Is the number of homologous exon splicing events higher than would be expected?

We previously found that substitution by homologous exons was among the least common annotated splicing events [24], so at first glance the number of homologous exon events detected in the human and mouse analyses is surprisingly high. In order to test whether there were significantly more homologous exon substitution (HES) events than expected in the human analysis, we hand-curated the results from BLAST [44] searches against the GENCODE 20 gene set (see Materials and Methods section) to generate a set of 157 genes with HES events that was independent of the events we detected in the proteomics experiment. We found peptide evidence for 33 of these 157 genes (21%) in our proteomics analysis. We carried out a Fisher test to determine whether the 21% detection rate for the HES genes was significantly different from the 0.01% AS event detection rate for the remaining 19,850 annotated genes: the p-value was close to zero (< 2.2e-16). The set of identified AS events is significantly enriched in homologous exon events.

### Is the enrichment in certain types of splicing caused by biases in the methods we used?

In pure theoretical terms, the greater the differences between the two isoforms, the easier it should be to identify peptides for both sides of the event and the fewer peptides we should need to detect that event. The easiest AS events to detect should be the longest substitutions (of any type), the largest indels and those cases where there are two completely different proteins. By way of contrast the hardest events to find ought to be substitutions that are almost identical, such as highly similar homologous exon events (there are events that change just a single residue) short indels, or NAGNAG splicing events (for NAGNAG-type splicing events, that generally result in a single residue indel, only a single peptide can identify each side of the splice event). Of course the detection of peptides is not totally random and there are other factors that influence, such as the number of lysines and arginines around the spliced region and how easy it is to detect the individual discriminating peptides by mass spectrometer. However, it is important to bear in mind that all these factors are rendered irrelevant if the protein isoform containing the peptide is not expressed in sufficient quantities to be detected in proteomics experiments.

It ought to be easier to find splicing events for those proteins that are more abundant. In order to identify a pair of splice isoforms for a gene, we need to detect at least two peptides for that gene. We binned genes by protein abundance (here protein abundance is measured as the number of peptides identified for each gene) and plotted the distribution of the AS genes against the background (the remaining annotated genes). As expected, there is a relation between peptide abundance in proteomics experiments and AS detection (see S8 Fig).

**Fig 3:**
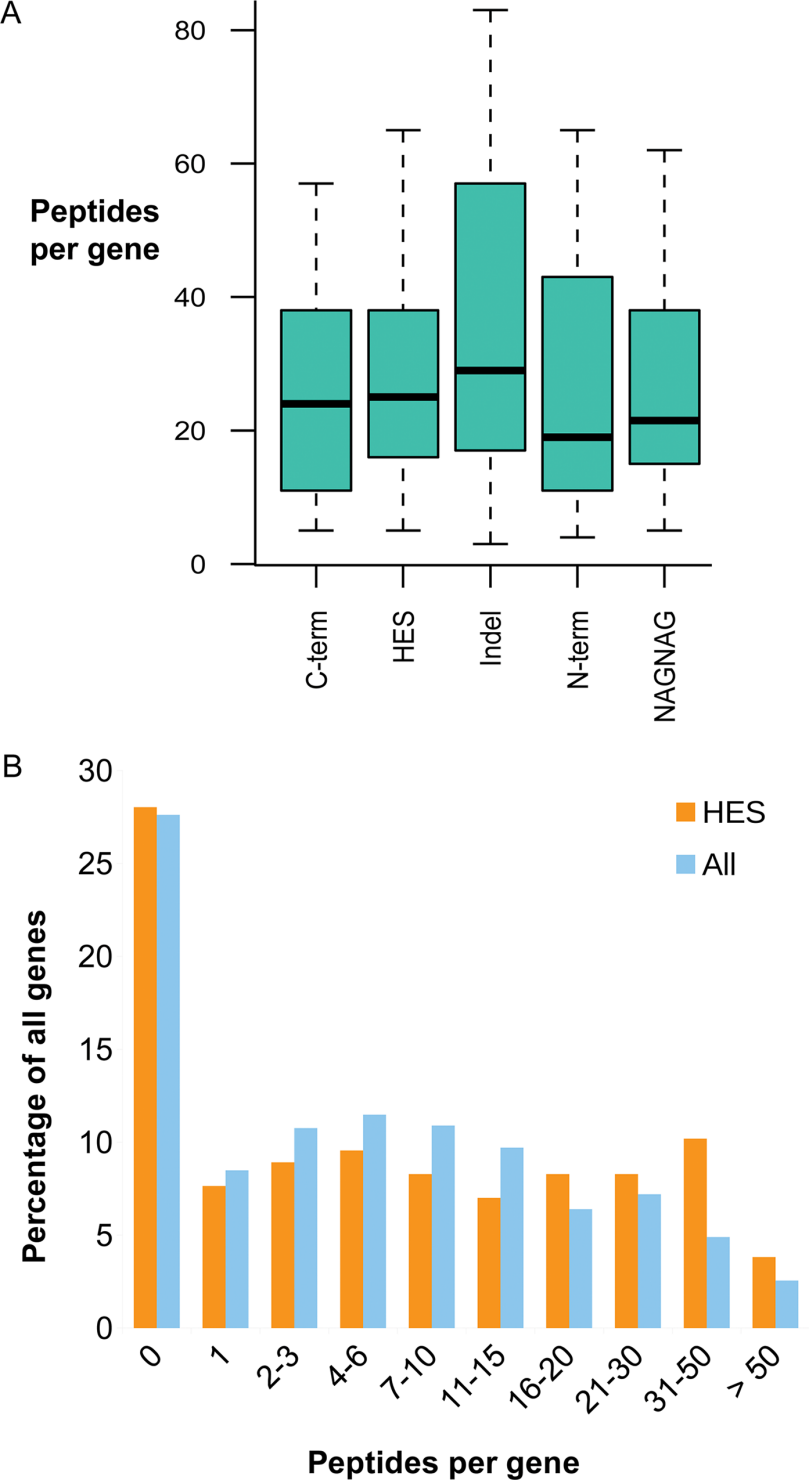
Numbers of peptides detected by ISE type. In A, boxplot of the five most common splice events (C-terminal substitutions, indels, homologous exon substitutions, N-terminal substitutions and NAGNAG-type splice events) showing the median (thick band) number of peptides per gene, the first and third quartiles (the bottom and top of the boxes). In B, genes binned by the number of peptides detected in the human experiments. Distributions are shown for the 157 genes with homologous exon substitutions (HES) that we identified with BLAST and for those genes that are annotated with at least one splice event (All).

If the detection of different types of splicing events is determined by purely theoretical considerations, we should need fewer peptides per gene to identify those splice events that are easier to detect (two distinct proteins) and require more peptides per gene to identify those that are harder to detect (NAGNAG splicing events). However, it turns out that there are few significant differences in the numbers of peptides identified per gene for each of the different splice types (Fig 3A). Wilcoxon rank tests between each group show that the only significant differences were between the “two protein” type and the other types (fewer peptides were needed to detect two distinct proteins as ought to be expected), and between the C-terminal substitution events (fewer peptides) and indel events. We detected substantially fewer peptides in genes in which we identified NAGNAG-type splicing events than in genes where we identified indels when would expect to need to identify more peptides per gene to identify NAGNAG events. Even though NAGNAG splicing events are theoretically the hardest splicing events to confirm, 13.5% of the alternative splicing events we detected were generated from NAGNAG splicing.

We can infer from these results that there is not a strong bias in protein abundance towards any one of different types of splicing event, which implies that the abundance of HES within the set of detected AS events (ISE) is not due simply to their being easier to detect.

We next wanted to determine whether the enrichment of homologous exon events was simply because they are mostly found in highly expressed genes. In principle the results from our study suggest that this bias is unlikely; if we were detecting HES events only because they were in highly expressed genes, we would also expect to detect evidence for other events in the same genes (HES genes are all annotated with other types of splice event in addition to the HES event). In fact we only detected non-HES splice events in one HES gene, *LDB3*.

To test this we used the 157 HES genes identified with BLAST. We compared the numbers of peptides detected for these genes to a background set of the 13,157 genes that are annotated with multiple splice isoforms. We used these counts to bin the genes according to the number of peptides that were detected and compared the proportions for each of the two populations of genes (Fig 3B).

The two distributions (HES genes and background) are very similar, though there are a slightly higher proportion of the most highly expressed genes in the HES gene population. However, a t-test (two tailed, unequal variance, p = 0.191) shows that this is not significant. Hence, the enrichment of HES events among the AS events we identified is clearly not because HES genes are all highly expressed.

Nor is the enrichment in HES events a consequence of combining the results from the eight analyses—the proportions of each type of splicing event are similar in all eight individual analyses as can be seen in S3 Table and S9 Fig. However, we do find that the HES events that we identified are generally more ubiquitous than the other major splice event types; 36 of the 114 events identified within at least four of the individual analyses (31.6%) were HES events, while 34 were indels and just 12 were C-terminal substitutions (S10 Fig).

These results suggest that the abundance of homologous exons in the alternative splice isoforms identified at the protein level is a real biological phenomenon, and not an artefact of the methods employed in the analysis.

### Conservation of homologous substitution splicing events

We detected peptides that mapped to paralogous splice events in six human protein families. These splice events clearly predated gene duplication in these families and have remained conserved in at least some of the paralogous genes that make up the family members. We identified homologous events for members of the enigma (*LDB3*/*PDLIM3)*, alpha-actinin (*ACTN1*/*ACTN4)*, dynamin (*DNM1*/*DNM2)*, plasma membrane calcium-transporting ATPase (*ATP2B1*/*ATP2B4*), reticulon (*RTN3*/*RTN4)*, and tropomyosin (*TPM1*/*TPM2*/*TPM3*/*TPM4*) families. According to EnsemblCompara 78 [45], the gene duplication events date back to vertebrates in the case of the tropomyosins, reticulons and alpha-actinins and jawed vertebrates in the case of the dynamins and the plasma membrane calcium-transporting ATPases. In the case of the enigma family the duplication dates back to chordata phylum. The origin of the splice events we identified must have pre-dated these duplication events. This strongly suggests functional relevance for the splicing events in these genes. The alternative isoforms we detected in five of the six families (enigma, alpha-actinin, dynamin, the plasma membrane calcium-transporting ATPases and tropomyosin) were generated from mutually exclusive homologous exons.

Since five of the six paralogous splice events were generated from HES events, we looked further into the evolutionary conservation of mutually exclusive homologous exons. We scanned the genomes of five distantly related aquatic vertebrates to identify long-standing conservation. We chose lamprey, spotted gar, fugu, zebrafish, and coelacanth as target species to date the origin of each splicing event. We found that every one of the 60 homologous exon splicing events that we identified in the proteomics analysis were present in at least one of these species, implying that they evolved in the ancestor jawed vertebrates or earlier, at least 460 million years ago [46].

As a comparison we calculated the proportion of alternative exons annotated in GENCODE 20 that were conserved between human and mouse. We found that just 19.3% of these alternative human exons were conserved in mouse. Human and mouse diverged close to 90 million years ago, so there is a notable difference in conservation between the HES events we identified and those annotated in the genome.

In a previous paper we showed that the older and more conserved a gene, the more likely we were to identify it in proteomics experiments [39]. The same seems to be true here—the splice isoforms that we identify in proteomics experiments, in this case the isoforms generated from homologous exon events, are the oldest, most conserved splice isoforms.

### Tissue-specific expression of alternative isoforms

The analysis of peptide data from the Kim [33] experiments allowed us to investigate tissue-specific alternative splicing. Tissue or cell specific alternative splicing is difficult to detect. Many splice events can only be identified with a single peptide and if that peptide is only detected sporadically, its absence from a tissue does not necessarily imply that it is not expressed in that tissue. Ideally each experiment should have a number of replicates to increase the probability of catching a hard-to-detect peptide. In this regard the Kim study was particularly useful, since most tissues had a small number of replicates.

The evidence for tissue-specific expression of splice isoforms principally came from four tissues: foetal and adult heart, adult cortex and foetal brain. This is not because these tissues had the most peptides (while foetal heart found the most peptides, the other tissues found the 8th most, the 22nd most and the 24th most peptides). We found five tissue-specific isoforms in brain. *FYN* is a tyrosine protein kinase that has two isoforms generated by homologous exons. Isoform 1 (FYN-B) and 2 (FYN-T) are supposed to be highly expressed in the brain and in hematopoietic cells, respectively [47]. The Kim experiment identified tissue-specific splicing of *FYN* [33] and we confirmed that FYN-B was indeed present in adult and foetal brain samples, while there was evidence for FYN-T in blood cells. *FYN* regulates cytoskeletal remodelling and cell survival by phosphorylating a number of proteins. Curiously we identified brain-specific splice isoforms for two of these: *MAPT* (Tau protein) and *AGAP2*. Isoform 1 of *AGAP2* (PIKE-L) is known to be brain specific [48] and we found peptides for this isoform in adult and foetal brain in the Kim experiment. Isoform 2 (PIKE-A) is described as ubiquitous: we detected peptides in blood cells. *MAPT* isoform PNS-tau is expressed in the peripheral nervous system while other isoforms are expressed in the central nervous system [49]. We found peptides for PNS-tau in adult heart, while peptides mapping to other *MAPT* isoforms were found in both adult and foetal brain. The splicing events that distinguish the *MAPT* and *AGAP2* isoforms are indels. We also found a brain specific alternative isoform for *GLS* (gelsolin) with different N-terminals, and heart and brain-specific isoforms for *VDAC3* (Voltage-dependent anion-selective channel protein 3), a mitochondrial membrane protein with a NAGNAG splicing event (Fig 1).

**Fig 4:**
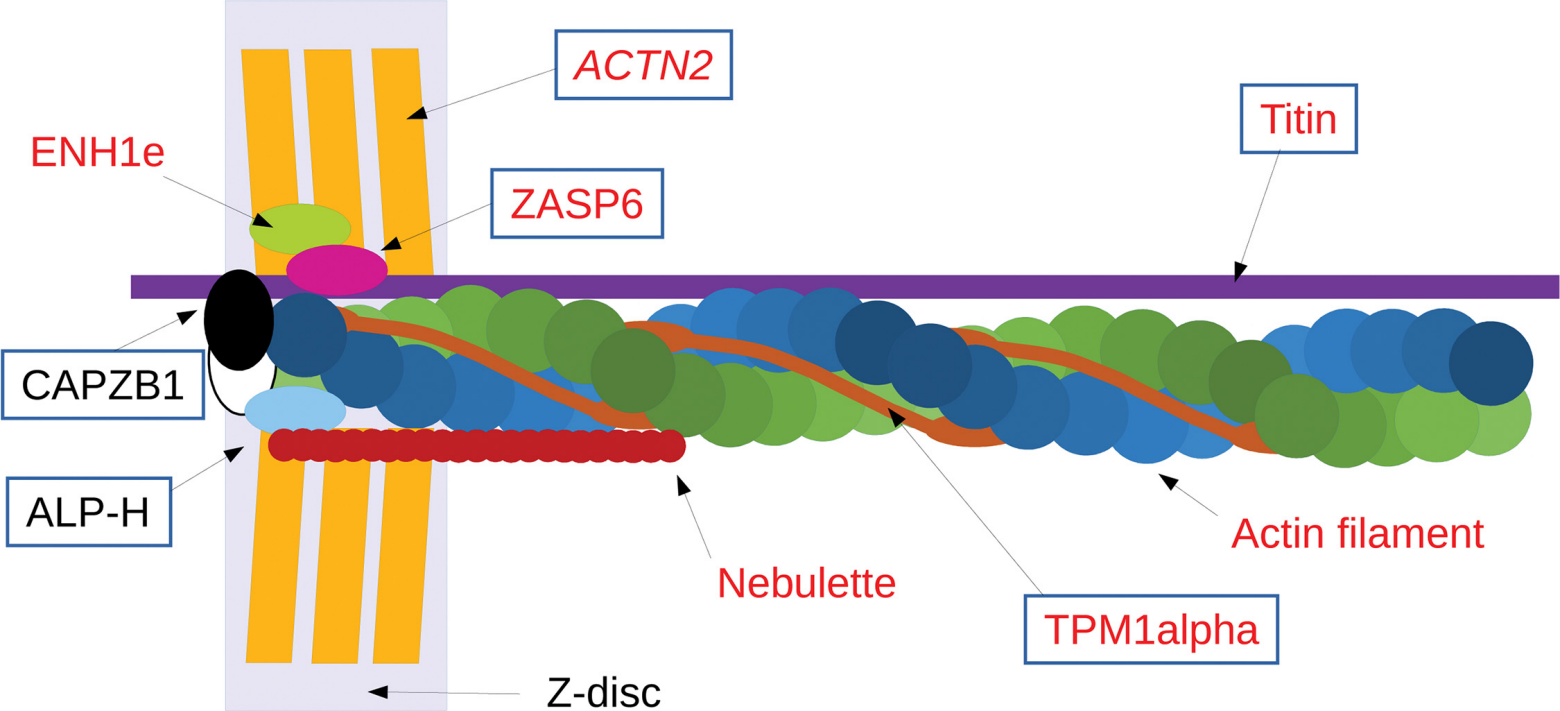
Cardiac specific splice isoforms. A general schema showing the possible location of the heart-specific splice isoforms identified in the Kim data set and their relation to the organization of actin in the thin filaments and Z-discs. Splice isoforms are shown for the following genes: *NEBL* (nebulette), *TPM1* (TPM1alpha), *CAPZB* (CAPZB1), *LDB3* (ZASP6), *PDLIM3* (ALP-H), *PDLIM5* (ENH1e) and *TTN* (Titin). The representation also includes alpha actinin 2 (*ACTN2*) the main component of the Z-discs. *ACTN2* is also annotated with homologous exons, but we did not identify peptides for the distinct isoforms. Alternative isoforms implicated in DCM are highlighted in red, while those generated from homologous exons are boxed.

The other hotspot for tissue-specific splicing was the heart. We found heart-specific splice isoforms for a further nine genes and most of these genes locate to the thin filaments or Z-discs (see Fig 4). Peptides from the Kim analysis identify cardiac specific splice isoforms for three members of the ALP/enigma family [50]. All seven family members are cardiac expressed, but specific functions have only been found for *PDLIM3* (ALP), *PDLIM5* (ENH) and *LDB3* (ZASP). All three are present in the Z-discs and interact with *ACTN2*, a gene that is estimated to make up 20% of the *Z*-disc mass [51]. *LDB3* and *PDLIM5* have both been implicated in dilated cardiomyopathy DCM [50,52]. The data from the Kim analysis suggested that ZASP1 is the major *LDB3* isoform expressed isoform in foetal heart, while ZASP2 and ZASP6 seem more highly expressed in adult heart. Isoform ZASP1 differs from isoforms ZASP2 and ZASP6 by the substitution of a remotely homologous exon. The two isoforms of *PDLIM3* we identified in the analysis (ALP-SK and ALP-H) are formed from a splicing event paralogous to the one in *LDB3*. The peptides from the Kim analysis locate ALP-H to adult and foetal heart and ALP-SK to oesophagus. The third gene, *PDLIM5*, has a heart-specific isoform (ENH1e) that differs from the non-cardiac isoform by a large insertion.

*NEBL* is one of the very few genes that generate splice isoforms with different Pfam domain compositions. The main isoform, nebulette, contains 14 nebulin repeats and a C-terminal SH3 domain. The shorter isoform LIM-nebulette swaps 12 of the N-terminal nebulin repeats for a LIM domain. Nebulette is a cardiac-specific isoform that is known to bind actin at the Z-discs [53]. At least four nebulette-specific amino acid variants [54] have been implicated in DCM. The data from the Kim analysis locates nebulette uniquely to adult and foetal heart tissue, while LIM-nebulette was found in frontal cortex, spinal cord, lung, kidney and prostate.

*TPM1* (tropomyosin) is one of just nine human genes that is annotated with multiple HES events. It is closely involved with nebulette in heart muscle and their interaction is important in the maintenance and stability of the thin filaments [55]. Again *TPM1* is known to be important in DCM; a number of *TPM1* single nucleotide variants that are likely to be pathological for DCM have been described in the literature [56]. We mapped these variants to the *TPM1* splice isoforms and found that all twelve likely pathogenic variants mapped to isoform TPM1-002 (TPM1alpha) suggesting that this was the most important isoform in heart tissue. Data from the Kim analysis confirmed that TPM1alpha is preferentially expressed in heart tissues [57]. We identified more peptides for TPM1alpha than any other *TPM1* isoform in every single one of the nine heart tissue experiments and fewer peptides for TPM1alpha in all other tissues.

*CAPZB*, the beta subunit of F-actin-capping protein, generates two proteins via alternative 3’ homologous exons that localize to different regions in the heart [58]. Splice isoform CAPZB1 is located at the Z-discs where it caps the thin filaments (see Fig 4). We found more evidence for CAPZB1 in heart tissues in the Kim analysis, while the CAPZB2 isoform was identified in liver, kidney, foetal brain and all red blood cells. We also identified a known cardiac-specific isoform for *TTN* (cardiac novex-3) and possible heart-specific isoforms for *TPM2* and *ITGA7*, both of which are known to be present in the heart but which are not annotated with cardiac-specific isoforms. The isoforms for all three of these genes were generated by HES events.

In total we found evidence for tissue-specific alternative splicing for 14 genes. Many of these genes are known to interact with each other. *FYN* interacts with *MAPT* and *AKAP2* in the brain, while seven of nine heart-specific isoforms are located to the Z-discs and at least five are known to be important in DCM (Fig 4). Though this is only a small sample, it is interesting to note that there seem to be tissue-specific protein-protein interaction networks involving AS isoforms. More than half of these 14 genes (and 7 of the 9 genes with cardiac specific isoforms) generate their tissue specific splice isoforms via alternative splicing of homologous exons.

### Alternative splicing and protein structures

Very few structures of alternative isoforms have been crystallised in the PDB structural database [59]. Hegyi and colleagues [60] found just 15 cases with resolved 3D structures of splice isoforms in the PDB. It has been suggested that many splicing events (in particular non-conserved splicing events) will result in unstable conformations [61]. This may explain the lack of crystallised structures: proteins that do not fold in stable conformations will not form proper crystals and their structures will not be resolved.

We looked for evidence of protein 3D structures for all 282 splice events detected in the analysis in the PDB, allowing the splice isoforms to come from any related species. We found ten pairs of alternative isoforms in which each isoform had a resolved 3D structure. Nine of the ten pairs were generated from homologous exons (Table 1), suggesting that structures generated from homologous exons might be easier to resolve. Among the 157 HES genes identified by BLAST searches, we found another 9 pairs of structures for alternative isoforms generated from homologous exons making a total of 18 pairs of structures for isoforms generated from homologous exons deposited in the PDB.

**Table 1:** A list of genes with pairs of alternative isoforms that were identified with peptides in the analysis and that have resolved PDB structures.

Gene	PDB Id 1	PDB Id 2	Type	Notes
*ACP1*	1xww	3n8i	Homologous	
*CDKN2A*	1d9s	1hn3*	Two Proteins	*Mouse
*DNM1*	4uud	3zvr*	Homologous	*Rat
*H2AFY*	1zr5	2fxk	Homologous	
*KHK*	3b3l	2hqq	Homologous	In Hegyi et al [60].
*MAPK14*	1r39	3oht*	Homologous	*Salmon
*MASP1*	4kkd	4igd	Homologous	
*PFN2*	1d1j	2v8c*	Homologous	*Mouse
*PKM*	3srf	3srd	Homologous	
*SNAP25*	1jth	1sfc	Homologous	

**Fig 5:**
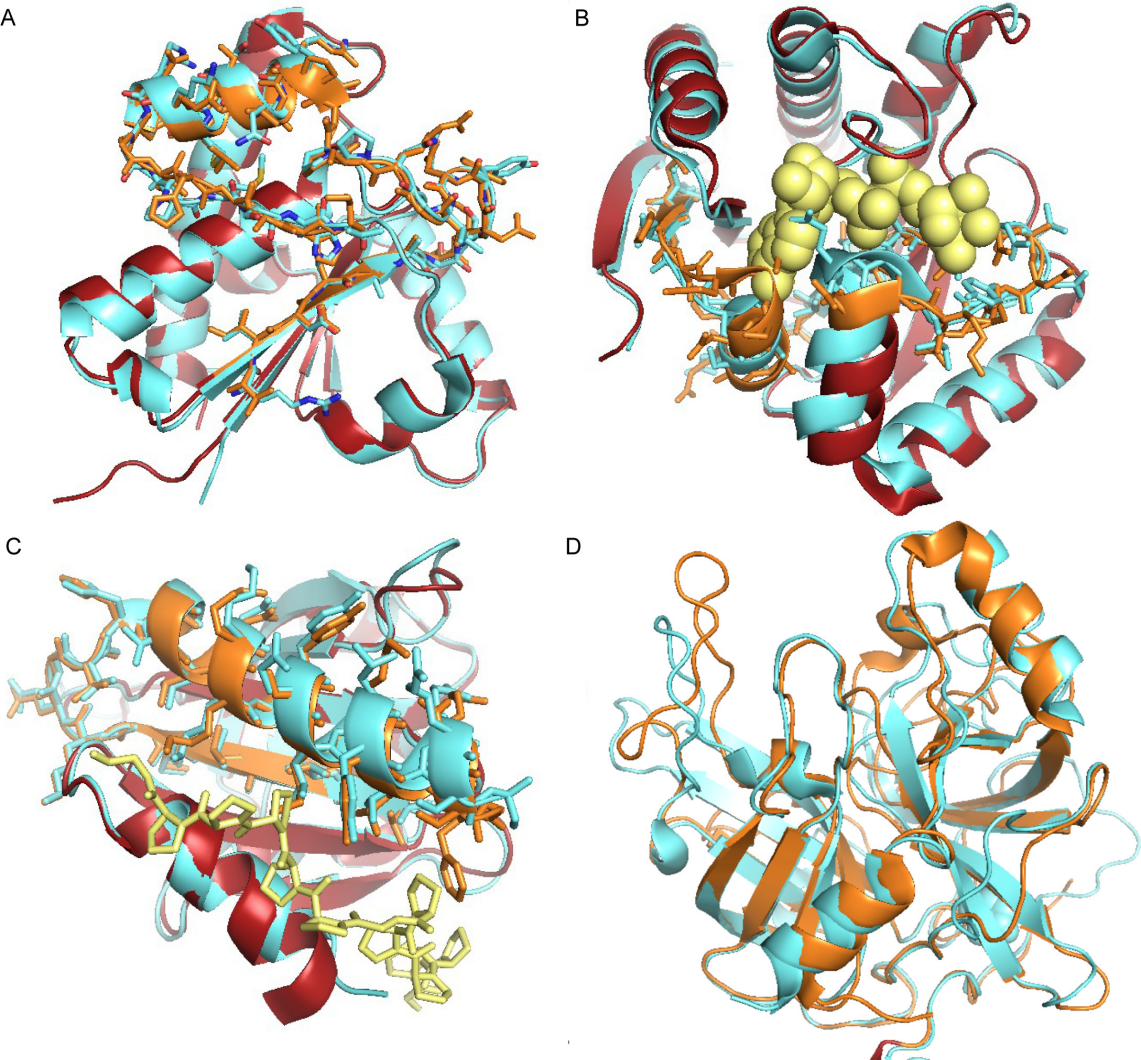
Resolved structures of homologous exons specific splice isoforms. A. Two isoforms generated from homologous exons for the gene *ACP1*, superimposed structures in red and cyan, regions generated from homologous exons in orange and cyan and side chains shown as sticks; B. Two isoforms generated from homologous exons for the gene *H2AFY*, superimposed structures in red and cyan, regions generated from homologous exons in orange and cyan and side chains shown as sticks, the nucleotide substrate is shown In yellow spacefill, the orange residues would clearly clash with the ligand; C. Two isoforms generated from homologous exons for the gene *PFN2*, superimposed structures in red and cyan, regions generated from homologous exons in orange and cyan and side chains shown as sticks, the proline rich peptide is shown In yellow; the orange residues come from a mouse protein; D. Two isoforms generated from homologous exons for the gene *MASP1*, superimposed structures in red and cyan; for this gene the whole trypsin domain is generated from homologous exons. All images of protein structures generated using the PyMOL Molecular Graphics System, Version 1.7 Schrödinger, LLC.

The structures of the 9 pairs of alternative isoforms identified in our analysis are shown in Fig 5 and S11, S12, S13, S14 and S15 Figs. The HES events have a range of effects on the structures. The HES region of the gene *ACP1* is confined to one surface of the protein, while the homologous substitution in gene *H2AFY* will clearly affect the binding to the nucleotide substrate. In gene *PFN2* the homologous region (from the orthologous mouse protein, but 100% sequence identical) may affect binding to the proline rich peptide substrate. In gene *MASP1* the whole trypsin domain is generated from homologous exons that have less than 33% identity, but the two proteins maintain the same fold.

The two structures resolved for the *MASP1* gene illustrate an important principle regarding the effect of alternative splicing on protein structures. It is clear from Fig 5D that the overall structure of the alternative *MASP1* trypsin domain is not affected even though the identity between the two alternatively spliced trypsin domains is only 33%. In fact this is to be expected since protein structures are very stable in the face of evolutionary change. Two proteins as little as 10% identity can have the same overall structure, as long as they are homologous [62]. By way of contrast indels and non-homologous substitutions that fall in regions with globular may produce completely different folds (or unfolded proteins) even though the two proteins have a relatively high identity over their whole structure.

The final pair of resolved alternative isoforms comes from the well-studied *CDKN2A* gene where translation from two different (conserved) frames results in two completely different proteins (S16 Fig). There are many structures for isoform p19INK, but just one for p16ARF and this structure is from mouse.

### Splicing favours intact Pfam domains

We looked at the effect of splicing events on the domains annotated in the Pfam functional domain database [63] for the 282 splicing events we identified. Pfam A domains were mapped to all isoforms with the program Pfamscan [63]. We counted a Pfam domain as broken if the splice event would cause the domain to lose or gain five or more residues.

The alternative splicing events identified in this study tended to not to have an effect on the composition of Pfam functional domains. Just 19 of the 282 splice events identified in the proteomics analyses (6.7%) would break Pfam domains, while 20 more (7.1%) would lead to the loss of one or more Pfam functional domains (while leaving the remainder intact). Five ISE would result in a swap of one set of Pfam domains for a different set. The remaining 84.4% of identified splice events would leave Pfam domains untouched. In the case of the homologous exons where the HES event coincided with a Pfam domain, we considered the Pfam domain unbroken, except for the plasma membrane calcium-transporting ATPases (*ATP2B1*/*ATP2B4*), where the substitution event clearly broke a poorly defined Pfam domain (see S17 Fig).

**Fig 6:**
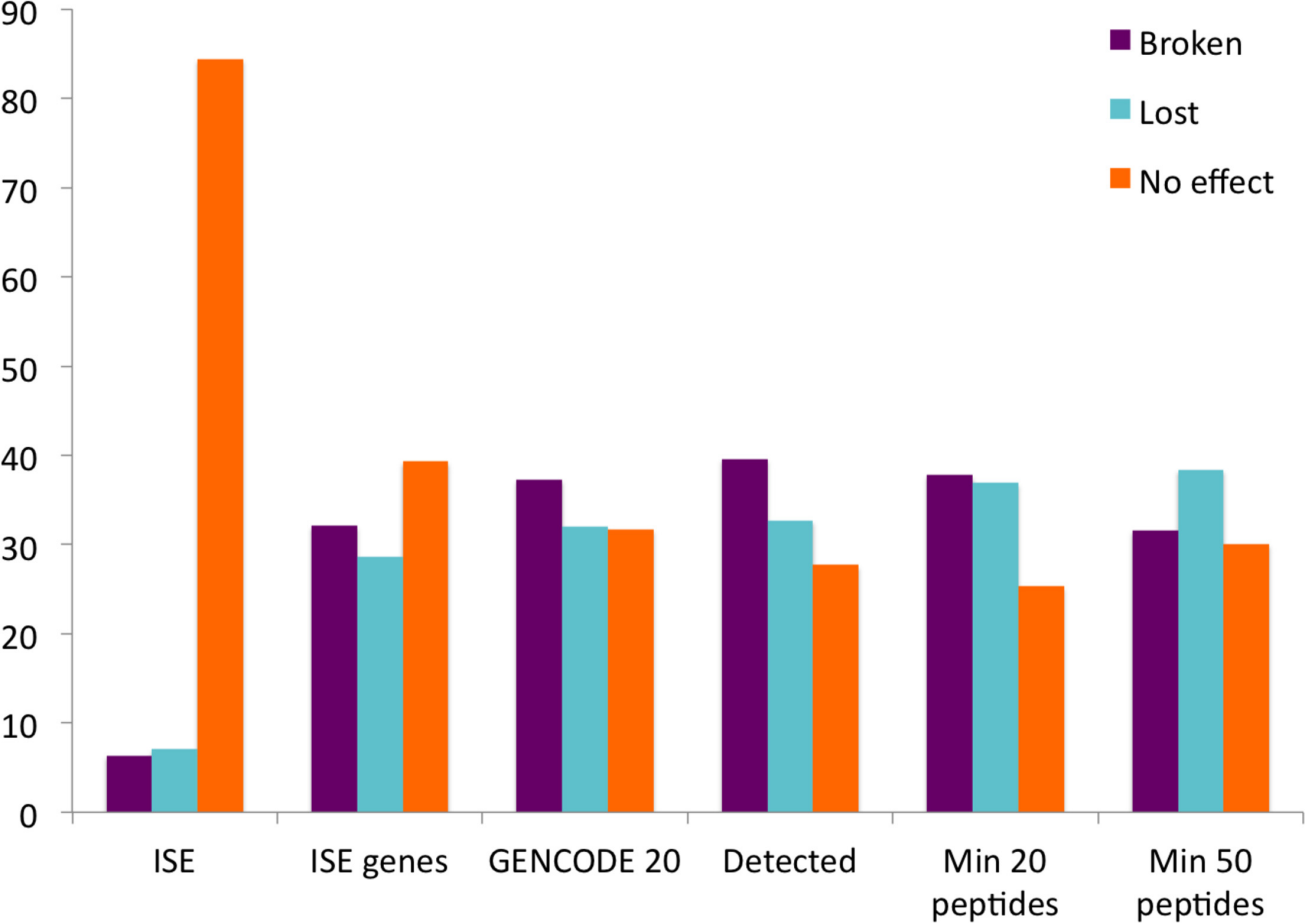
The effect of splice events on Pfam functional domains. The percentage of splice events that would produce alternative isoforms with a damaged Pfam domain (Broken), that would cause the alternative isoform to lose a whole Pfam domain (Lost) and that would have no effect on the Pfam domain composition of the alternative isoform (No effect). Percentages shown for six sets of events, all events annotated at the transcript level for all protein coding genes in the GENCODE 20 annotation of the human genome (GENCODE 20), all events annotated at the transcript level for all genes identified in the proteomics analysis (Detected), all events annotated at the transcript level for those genes for which we identified at least 20 peptides (Min 20 peptides), all events annotated at the transcript level for those genes for which we identified at least 50 peptides (Min 50 peptides), all the splice events annotated at the transcript level for the 246 genes in which we identified splice events (ISE genes), and the 282 splice events that we identified peptides for from those 246 genes (ISE).

We calculated the effect of splicing events on Pfam domains over the whole GENCODE 20 gene set and against 4 other subsets of genes as a comparison. We took the principal isoform for each gene from the APPRIS database [64]. The APPRIS principal isoforms have been shown to be a reliable means of predicting dominant isoforms at the protein level [18]. We then generated pairwise alignments between these principal isoforms and all alternative isoforms. We counted all unique splice events from the pairwise alignments between the principal isoform and each of the alternative isoforms for all genes. We mapped the splicing events to the Pfam-A annotations from Pfamscan. The four subsets of genes compared were: all 12,716 genes we detected peptides for, all genes that we detected at least 20 peptides for (2,271 genes, the median number of peptides for the HES genes), all genes that we detected at least 50 peptides for (385 genes), and all the 246 genes for which we detected splice isoforms (Fig 6).

The GENCODE 20 gene set annotates 45,346 unique alternative splicing events; 37.3% of the splice events (16,937) occur inside Pfam functional domains and would generate alternative isoforms with damaged functional domains. Another 32% (14,766) of the splicing events would lead to the loss of one or more whole Pfam domains (Fig 6). The figures were similar for the other four gene subsets (Fig 6), even for the subset of 246 genes for which we detected splice isoforms. The 246 genes that we identified splice isoforms for are annotated with 1,650 splice events, 524 (31.8%) would split Pfam domains, while 477 (28.9%) would lead to the loss of one of more whole Pfam domains (Fig 6). In clear contrast, the splice events that we detected in the proteomics experiments tended not to lead to the loss or damage of Pfam functional domains. These results clearly show that most alternative isoforms with damaged or lost Pfam domains are not produced in quantities detectable in standard proteomics experiments. This strongly suggests that many variants that affect Pfam composition are produced in very low quantities or that there is some form of control at the level of translation, or post-translation, that protects the cell against protein isoforms with damaged domains.

### Do we find fewer splice events than would be expected?

While we find peptides for 12,716 genes, we identify a mere 282 splice events (0.62% of the annotated events). Over the 8 experiments we identified just 0.02 alternative splice events per identified gene while the numbers of annotated splice events per gene is 2.28. This seems to be a very small proportion.

Not only do we identify very few splice isoforms, we also detect very few alternative peptides. If we were to use trypsin to digest the human proteome (in this case the GENCODE 20 annotation of the human coding genes that we used to map the peptides from the eight analyses), we would generate 693,324 unique tryptic peptides of at least seven residues. A total of 11.21% of these 693,324 unique tryptic peptides would come exclusively from isoforms tagged as “alternative” by APPRIS (those isoforms that were not principal isoforms, see Materials and Methods section). In contrast in our analysis just 0.38% of the 149,612 discriminating peptides that we detected mapped to alternative isoforms. The equivalent numbers for the alternative peptides, splice events and genes identified in the individual experiments can be found in the supplementary S1 Table.

We attempted to estimate the number of AS genes we would have expected to identify from our data using a range of simulated null models. For the first model we generated tryptic peptides from the GENCODE 20 gene set by *in silico* lysis. Each peptide was represented just once in the database of peptides. We used just tryptic peptides for this simulation because all possible missed cleavage combinations would make the search space too large. For this first model we did not eliminate any redundancy from the GENCODE 20 annotation, so as to approximate a model in which all annotated transcripts were expressed equally. If we had only used tryptic peptides in our experiments we would have found evidence of alternative splicing for 226 genes (20 of the AS genes were identified via missed cleavages), while there would have been evidence of three or more isoforms for 14 genes.

To simulate the expected numbers of AS genes we drew peptides at random from this database. In order to approximate the expression levels from the experiments, the number of tryptic peptides drawn for each gene was the same the number we identified in our analysis. We repeated the simulation 100 times and took the mean value to be the estimation of the number of AS genes we would have expected to find if genes were expressed at experimental levels and all isoforms of each gene were expressed equally.

The numbers of AS genes from this *in silico* analysis were substantially larger than those in our experiment; in our model we found evidence of alternative splicing for 3,508 genes (15 times greater than the experiments), and evidence of at least three isoforms for 937 genes (67 times greater than the experiments).

We repeated the simulation, but this time carrying out an *in silico* analysis that produced 50-times more peptides for the main isoform of each gene than for all the other isoforms. For this model we used the principal isoforms from the APPRIS database as a stand-in for dominant splice isoforms [64]. Again for each gene we randomly selected exactly the same number of tryptic peptides as we had identified in our experiment. We repeated the simulation 100 times. This simulation approximates a model in which genes have expression levels similar to the experimental expression and all peptides are equally detectable, but in which one isoform is produced 50 times more than the other isoforms. The simulation showed that we would have expected to detect 1,289 genes with evidence of AS at the protein level and another 152 genes with at least three distinct splice isoforms in this model. Again this is in clear contrast with the observed results and suggests that the majority of alternative isoforms may be produced at levels that are considerably lower than 2% of those of the main isoform. This evidence clearly supports a model in which most genes have one dominant isoform at the protein level [18].

## Discussion

We generated a highly reliable set of peptides from eight large-scale proteomics analyses by applying rigorous filters. The rigorous quality controls on the peptide data allowed us to be confident that the isoforms we identified were expressed and present in high enough quantities to be detected in proteomics analyses. With these peptides we detected the expression of alternative isoforms for 282 distinct splice events from 246 distinct human genes. While the filters undoubtedly limited the number of splice events we detected, they do mean that this set of alternative splice isoforms can be regarded as a gold standard for what can be detected in large-scale proteomics experiments.

Even with peptide data from eight large-scale analyses that cover a wide range of tissues, cell lines and developmental stages, we still detect many fewer alternative isoforms than would be expected from transcript data. We found peptides for almost 64% of annotated protein coding genes, but identified less than 0.6% of the annotated alternative splice events. In part this may be due to proteomics technology. Standard MS experiments generate relatively low coverage of the proteome and cannot detect peptides expressed at very low levels. This is a technical problem that is unlikely to be resolved in the short term.

We found unexpectedly high numbers of isoforms generated by alternative splicing of homologous exons; more than 20% of the splice events we detected in the human proteomics experiments came from mutually exclusively spliced homologous exons and these homologous substitutions made up 60% of the orthologous splicing events detected in both mouse and human experiments.

A surprisingly high proportion of isoforms from homologous exon substitutions had resolved 3D structures. The explanation for this may be simply that structures generated from homologous exons are easier to crystallize [61]. Homologous structures will maintain their 3D fold, while non-homologous exons that fall in structured regions may cause the 3D structures to become partially unfolded and therefore difficult to resolve.

The recent publication of a large-scale tissue-based proteomics analysis with replicates [33] allowed us to carry out a study of alternative splicing at the level of tissues. We found evidence for tissue-specific expression of fourteen pairs of alternative isoforms. Curiously those genes for which we detected tissue-specific splicing isoforms had at least one isoform that was specifically expressed in either heart or brain and many of them are known to interact. The heart-specific isoforms were particularly interesting because the majority proteins coded by these variants are known to locate at the Z-discs and to be involved in dilated cardiomyopathy. For many of the remaining isoforms the data was inconclusive. Seven of the nine heart-specific isoforms we identified were generated from homologous exon splicing events.

In 2001, Kondrashov and Koonin [65] found evidence for 50 genes with homologous exon substitutions across a range of species. They reported that half of the 29 HES for which they identified an ancestor arose in the mammalian lineages. With the data now available, we find that all the HES detected in our experiments (and 27 of the 29 HES identified by Kondrashov and Koonin), had their origins in the ancestor of jawed vertebrates or earlier, more than 460 million years ago. This is a remarkable level of conservation for alternatively spliced isoforms.

In contrast Modrek and Lee [66] found that only 25% of what they termed “minor” alternative exons were conserved between human and mouse. We carried out our own analysis of alternative exons (see Materials and Methods section) and found a similar result, just 19.3% of the 3,626 alternative exons we analysed (excluding homologous exons) were conserved between human and mouse. The homologous exon substitution events we identified are clearly much more conserved than this.

The ancient conservation of isoforms generated from homologous exon substitution events, taken together with the abundance of peptide evidence for these isoforms, their tissue-specific expression, and the fact that these events have a demonstrably subtle, non-disruptive, effect on protein structure, strongly suggests that alternative splice isoforms generated from mutually exclusively spliced homologous exons are likely to have important cellular roles that merit further investigation.

Most of the splice events we identify in this analysis would have relatively modest effects on protein structure and function; many alternative isoforms were generated from homologous exons and even most indels were either short or fell in regions that are likely to be unstructured. In fact very few of the splice events we detected would damage or cause the loss of conserved Pfam functional domains. This is in sharp contrast to the splice variants annotated in the GENCODE gene set, where the majority of the splice events would be expected to have an effect on Pfam domains.

The preference for splice events that do not disrupt Pfam functional domains and the analysis of evolutionary conservation strongly suggest that not all annotated alternative transcripts will be converted into stable proteins. One possible explanation for this finding is that alternative protein isoforms with damaged or lost functional domains are more likely to have a disruptive effect on cellular processes and their production may be subject to regulation by one of the many cellular quality control pathways [67–70], to ensure that isoforms with damaged domains are not present in the call in large quantities.

At the moment, it is still not clear how much of the alternative splicing observed in the transcriptome is functionally relevant. Our results suggest that, at the protein level at least, the diversity generated by alternative splicing may be smaller than most previous estimates. If true these findings will have important practical implications for variant-calling analyses that include potentially non-relevant transcripts [71] and will affect our understanding of how organisms and complexity evolve.

## Materials and Methods

The peptides for the human analysis came from eight proteomics datasets. Six of the peptide datasets came from large-scale experiments [32,33, 36–39]. The remaining sets of peptides came from two spectra databases, NIST (http://peptide.nist.gov/) and PeptideAtlas [21]. The peptides for the mouse analysis came from the same two mass spectrometry databases (NIST and PeptideAtlas). In addition we generated a set of peptides in house from an X!Tandem [72] search against spectra from mouse mass spectrometry experiments deposited in the GPM [28] and PeptideAtlas databases, following the protocol set out in Ezkurdia *et al*. [24].

Note that the nature of the PeptideAtlas and NIST databases means that a number of spectra from the experiments are likely to have been interrogated multiple times by different search engines and with different post-processing filters. The peptides that map to these spectra will be in our study when the peptide-spectrum mappings agree (mostly for reliably mapped peptides), but will not appear in our study when the peptide-spectrum mappings do not agree (which will often be the case for false positive mappings).

### Filtering for high quality peptide identifications

We wanted to make sure that the alternative isoforms that we detected were not identified from incorrectly mapped peptides, so we used a series of filters to remove as many false positive peptides as possible from each analysis. The peptides from the individual analyses were filtered as follows.

The peptides from the Geiger [36] and Nagaraj [37] experiments were treated in identical fashion. The peptides in these studies had a peptide false discovery rate (FDR) of 1%, but for the purposes of this experiment we required the peptides identified in the two datasets also to have an Andromeda [73] score of 100 or more. It has been shown that using multiple search engines increases performance [74,75] and since peptides identified by Andromeda with scores of 100 or greater are almost always in agreement with those identified by Mascot [76] for the same spectra [73], concentrating on the peptides with PSM above this score decreases the false positive rate.

The Kim experiment [33] used the Mascot and Sequest [77] search engines and the peptides identified in their analysis came from the union of these two search engines. The Kim experiment described the peptide-spectrum match (PSM) FDR as being 1%. We have shown that this is likely to be an underestimate [34]. In order to be more rigorous for the purposes of our experiment, we only included the peptides from the intersection of the two search engines used in the Kim analysis, that is, those peptides that were identified by both the Mascot and Sequest search engines.

The Wilhelm analysis [32] also used two search engines, this time Mascot and Andromeda. Again the peptides came from the union of the two search engines. The Wilhelm experiment had a 1% PSM FDR and a 5% peptide FDR, but again we found that this was likely to be an underestimate [34]. Upon analysis of the scores from the two search engines used we found a high number of dubious spectra in which Andromeda and Mascot agreed on a peptide match, but both search engines had very low scores. For the purposes of our experiment we treated the Wilhelm analysis in the same way as the Geiger and Nagaraj analyses; we only took those peptides identified with an Andromeda PSM score of 100 or more.

The NIST database uses five different search engines (Sequest, Andromeda, Mascot, X!Tandem and OMMSA [78]) to identify peptides from human spectra, and three search engines for spectra from mouse experiments. While the NIST database includes many peptides, the FDR is quite high. For the purposes of our experiment we filtered out those NIST peptide-spectrum matches identified by just one search engine.

The peptides from the Ezkurdia analysis [39] were identified with X!Tandem and had a peptide FDR of 0.1%, while the peptides from the Munoz [42] analysis were identified using Mascot and had a peptide FDR of 1%. PeptideAtlas peptides have a PSM FDR of 0.0002%.

All peptide data sets were then subject to the following filters: we filtered out non-tryptic and semi-tryptic peptides and only allowed peptides with missed cleavages that were supported by at least one of the fully cleaved sub-peptides. We applied the equivalent rule to the peptides from the Wilhelm analyses for peptides cleaved with LysC and chymotrypsin, and to the peptides detected in the Nagaraj analysis that were cleaved by GluC or LysC enzymes. In the case of the Ezkurdia, PeptideAtlas and NIST analysis we did not know the digesting enzyme *a priori*, so we chose the conservative option of assuming that all peptides were cleaved by trypsin. Search engines do not easily distinguish leucine from isoleucine due to their identical mass, so leucine and isoleucine residues were allowed to map to *either* leucine or isoleucine in the GENCODE20 gene set. All peptides that mapped to more than one gene were disregarded in the analysis. The numbers of peptides and genes identified in the individual experiments can be seen in S1 Table.

### Identifying the splice isoforms

We only mapped peptides that were identified by two or more different experiments or databases in our analysis of alternative isoforms. We excluded peptides identified in just one of the eight peptide data sets, since, even after the filtering carried out in this analysis, a proportion of these peptides are likely to be false positives.

For the human analysis the peptides were mapped to the protein isoforms annotated in the GENCODE 20 human gene set. The gene set was first filtered for pseudoautosomal genes and for read-through transcripts. Read-through transcripts are flagged in the GENCODE 20 annotation and are transcripts that are (generally) formed by skipping the last exon of a gene and reading through to the neighbouring gene or non-coding gene, or pseudogene. All these transcripts are highly unlikely to be translated into proteins and (most importantly) overlap with known coding transcripts. Read-through transcripts that overlap with coding transcripts would render these coding transcripts indistinguishable. The remaining GENCODE 20 gene set was annotated with 19,906 protein-coding genes and 92,341 protein isoforms; the manual GENCODE annotations are highly enriched in alternative isoforms [49]. 15,548 genes were annotated with more than one distinct splice isoform.

For the mouse analysis we mapped peptides to the isoforms annotated in the GENCODE mouse M2 gene set (equivalent to Ensembl74). The M2 gene set had 22,645 protein-coding genes and 51,610 transcripts. In the mouse gene set just 10,607 genes were annotated with protein sequence distinct variants.

We mapped isoform-discriminating peptides to the splice isoforms annotated in the GENCODE 20 gene set looking for peptides that mapped unambiguously to distinct splice isoforms. The initial set of splice isoforms was checked by hand by mapping the isoform-discriminating peptides to multiple alignments. Only those splice events for which we identified peptides that mapped to both sides of the events were included in the final set.

There was peptide evidence for the expression of 7 different isoforms from the UGT1A gene cluster. Although UGT1A transcripts are annotated as independent genes in GENCODE 20, we have treated them as splice variants of a single gene in this analysis. In the UGT1A gene cluster the individual genes/transcripts share a set of common 3’ exons and each has a unique (but homologous) 5’ exon (S1 Fig). As with the *Drosophila* gene *Lola* (where the variable exons are at the 3’ end rather than the 5’ end [79]), the different protein products are formed by joining one of a set of variable exons to the common exons,

The selection of the 5’ exons is thought to be determined via alternative promoters. The use of alternative promoters is not a standard alternative splicing mechanism, but the end result is the alternative splicing of exons, just as it is in *Lola* in *Drosophila*, another non-standard alternative splicing mechanism, where variable and constant exons are joined by *trans-* rather than *cis*-splicing. There are three other similar gene clusters in the GENCODE 20 gene set (UGT2A, PCDH-gamma and PCDH-alpha) and we regarded all four gene clusters as single genes with alternatively spliced protein isoforms.

### Identification of a set of human genes with mutually exclusive homologous exons

Based on Ensembl version 78 of December 2014 [40] we compared human transcripts to automatically identify a set of genes with mutually exclusive homologous exons. We defined the transcript with the longest amino acid sequence as the reference against which compare other transcripts and looked for pairs or sets of exons that are mutually exclusive, i.e. that do not co-occur in the same transcript. For exons that were more than 30 bps long, we obtained their amino acid sequences and compared them with BLAST v2.2.25 [44], setting an e-value threshold of 0.005. To validate each of the resulting potential homologous exons we assessed whether the exons occupied equivalent positions within the corresponding alternative transcripts. In addition, we discarded those cases in which one of the alternative exons belonged to a paralogous neighbour gene or pseudogene. Finally, we included additional cases of homologous exons that were identified by BLASTP searches [39], but missed in this automatic analysis. We visually inspected the alignments of all the potential cases. We ended with a set of 157 genes with (mutually exclusive) homologous exon substitutions. Although the proteomics analysis of alternative splicing revealed other additional cases of homologous exons beyond this set, we did not include them in this test set to avoid potential biases.

### Identifying homologous exons of ancient origin

To identify whether the homologous exons substitutions we detected were of ancient origin, here defined as those that originated more than 400 million years ago, we scanned the genomes of five distantly related vertebrates using TBLASTN [44] with the exons as amino acid query sequences, turning off low complexity filtering and setting an e-value threshold of 0.1. These five taxa included lamprey, spotted gar, zebrafish, fugu, and coelacanth, all of which were retrieved from Ensembl v75. We used bedtools v2.17.0 [80] to group sequence similarity hits based on the 95 percentile of gene lengths of each target species, then assigning these hits to annotated or new genes in each target species. For every target gene we determined whether it was orthologous or paralogous to the query human gene using EnsemblCompara phylogenetic trees [45]. Results were carefully revised to determine which genes from each target species had specific hits to each query exon, i.e. whether the human homologous exons were already present in that species. The origin of the homologous exons was inferred under the assumption that homologous exons have not been acquired independently in different species, i.e. we relied on Dollo parsimony [81].

### Determining the proportion of alternative exons conserved in mouse

We determined the constitutive exons to be those that were annotated as principal isoforms in APPRIS [64]. We defined as alternative exons all those protein-coding exons that did not overlap with the constitutive exons. We found 13,079 of these exons in the GENCODE 20 gene set. We improved the reliability of the annotation by filtering out those genes where the principal isoforms was not determined by the core modules or by unique CCDS identifier [82]. We further filtered these exons as follows.

We removed exons that were too short to identify homology with mouse in the TBLASTN searches. These were defined as those with a BLAST e-value higher than 0.001 when compared against the whole human proteome, which includes the query exons (these are exons for which we may expect a significant sequence similarity hit in the mouse genome if the exon is conserved). We also removed exons that were similar to exons in the APPRIS principal isoforms (e-value threshold of 0.1). This avoids complicating the interpretation of potential conservation and also excludes exons that can be defined as homologous. If exons overlapped with each other we took the longer of the exons as the representative. We also filtered out 15 genes presenting a large number of alternative exons caused by a gene model that was clearly not finished or had errors that were influencing the selection of the principal isoform by APPRIS (for example, *FRAS1*, where the gene model was unfinished in GENCODE 20 or *FIP1L1*, which had an unannotated read-through transcript that APPRIS selected as the main isoform).

After all the filtering steps, we ended up with a set of 3,626 alternative human exons. Using these exons as amino acid query sequences we searched the mouse genome with TBLASTN, turning off low complexity filtering and setting an e-value threshold of 0.1. We defined the exon as conserved when a significant similarity was found within a mouse gene (or close to the gene, we set a distance threshold based on the 95 percentile of gene lengths) that is present in the same EnsemblCompara phylogenetic tree than the corresponding human gene.

### APPRIS principal splice isoforms

APPRIS [64] derives a principal splice isoform for each gene based on the presence of protein features, such as protein structure, functional domains and cross-species conservation. These features can discriminate between splice isoforms because they have generally been conserved over large evolutionary timeframes. Isoforms that have “lost” these features are unlikely to be the principal isoform. APPRIS maps protein structural information from the structural homologs in the PDB [60] to individual isoforms, annotates functional information from the Pfam domain database [63] and from the functionally important amino acid residues from *firestar* [83] and evaluates the cross-species conservation of every isoform. The isoform with the most conserved protein features is chosen as the principal splice isoform. Where the APPRIS annotations are not sufficient to distinguish a single principal isoform, such as genes *TMPO* [84] or *MARVELD3* [85], APPRIS uses external annotations, such as presence in the CCDS database [82] in order to make a decision.

The variant chosen from the core APPRIS modules have been shown to be in almost complete agreement with the main proteomics isoform [18].

### The effect of splice events on Pfam domains

Here we selected a single isoform as the main isoform (using APPRIS principal isoforms) and defined an alternative splice event as being an event that changed the protein sequence between the main isoform and alternative isoforms. Alternative proteins that were simply truncated were not used to count splice events in order to avoid including annotated fragments of transcripts from unfinished gene models. We used PfamScan to annotate Pfam domains onto the transcripts and counted all those cases where the splice event would cause the Pfam domain to lose five or more amino acid residues (a damaged Pfam domain), or the whole Pfam domain (a lost Pfam domain).

## Supporting Information

S1 Table Breakdown of the data from the individual MS analyses. (PDF)

S2 Table Genes for which alternative isoforms were identified. (XLSX)

S3 Table Types of splicing events found in each experiment. The number of each type of splice event detected in each of the eight peptide data sets. The types of splicing events are described in the main paper. Splice events could be identified in the individual data sets with just a single peptide for each side of the event.
(PDF)

S1 Fig Splice event specific peptides for the UGT1A cluster. A. Multiple alignment of the N-terminal region of seven of the isoforms of the UGT1A cluster that were identified in the analysis. The peptides we detected in the eight data sets are mapped onto the alignment in colour. Although all peptides discriminate between some isoforms (even though they do not fall in the splice junction) red peptides discriminate between all splice isoforms, blue peptides only discriminate some of the isoforms. B. The UGT1A cluster exons.
(PDF)

S2 Fig The indel detected for gene *HNRNPC*. A portion of the gene model of HNRPNC from the Ensembl web page. The two isoforms 002 and 007 differ by the insertion/deletion of 13 amino acid residues at the N-terminal (5’ end) of the protein. Here the second exon is missing in variant 007.
(PDF)

S3 Fig Mutually exclusive homologous exons for gene *CALU*. In A, a section of the *CALU* gene model from the Ensembl web pages, with arrows showing the homologous exclusively spliced exons for variants 001 and 002. In fact there are three splice events shown in this image, the homologous substitution, an N-terminal extension, which comes from the extra exon at the 5’ end in variants 003 and 005, and which cannot be detected by proteomics data, and a C-terminal substitution that results from skipping the penultimate coding exon in 006 (a C-terminal substitution in protein terms since the frame of the last exon is changed by the exon skip event. In B part of the pairwise alignment between the protein sequences of variants 001 (ENST00000249364) and 002 (ENST00000449187) showing the similarity between the two protein sequences that results from the homologous splicing event. Homologous exon events can also occur with the 5’ and 3’ exons.
(PDF)

S4 Fig C-terminal substitution for *TMPO*. A section of the *TMPO* gene model from the Ensembl web pages. Here the large 3’ exon in variant 001 is replaced by five smaller coding exons in variants 002 and 014. The substitution is not homologous. *TMPO* was one of the genes with the clearest evidence of protein level alternative splicing; one of the alternative exons was derived from a transposon [84].
(PDF)

S5 Fig N-terminal substitution for *NEBL*. A section of the *NEBL* gene model from the Ensembl web pages showing the two most important isoforms, nebulette (004) and LIM-nebulette (003). Most if the 5’ exons of nebulette are replaced by just four exons in LIM-nebulette, though LIM-nebulatte does add a LIM domain.
(PDF)

S6 Fig *KIF23* internal substitution. A section of the pairwise alignment between two *KIF23* isoforms. The internal exon substitution results in a swap 100 residues for just six residues. There is no homology. These substitutions were very rare in the proteomics experiments.
(PDF)

S7 Fig *MICAL3* gene would give rise to two unique proteins. The Ensembl gene model for the *MICAL3* gene. Isoforms 001 and 002 would be translated from one set of overlapping exons while 011 and 017 would be translated from a different set of non-overlapping exons.
(PDF)

S8 Fig Histogram of peptides detected for AS and all genes. This histogram shows the peptide abundance for all genes with at least 1 peptide (in green) and the genes for which we detected evidence of alternative splicing (AS genes, red). As expected, alternative splicing detection is related to peptide abundance in proteomics experiments (which here is the measure of levels of protein expression); the more peptides we detect for a gene, the more likely we are to detect alternative splicing.
(PDF)

S9 Fig Types of splicing events found in each experiment. The percentage of each type of splice event detected in each of the eight peptide data sets. The types of splicing events are described in the main paper. Splice events could be identified in the individual data sets with just a single peptide for each side of the event. This is the figure version of supplementary S3 Table.
(PDF)

S10 Fig Splicing events found in multiple experiments. The percentage of each type of splice event for those splice events detected across multiple experiments. Splice events in the first column are those events that were detected in all eight experiments, the second column the events were detected in seven of the eight individual experiments, the third column in six individual experiments, and so on. The types of splicing events are described in the main paper. Splice events could be identified in the individual data sets with just a single peptide for each side of the event.
(PDF)

S11 Fig The superimposed structures of two isoforms of *DNM1*. The two homologous exons are shown overlapping in orange (PDB structure: 4UUD) and aquamarine (PDB structure: 3ZVR) with the side chains represented as sticks. The two structures have relatively large differences for structures produced from homologous exons. In part this will be because the one of the dynamin isoforms (3ZVR) was resolved for rat rather than human, and in part will be because helical structures are generally more flexible than beta-sheet based structures.
(PDF)

S12 Fig The superimposed structures of two isoforms of *KHK*. The two homologous exons are shown overlapping in orange (PDB structure: 3B3L) and aquamarine (PDB structure: 2HQQ) with the side chains represented as sticks. Interestingly the structural superposition allows us to make a hypothesis for a subtle change in function for between the two ketohexokinase isoforms. While the catalytic residues identified by *firestar* superimpose well, the ligand binding residues (shown in yellow) in the region of the homologous exon have a different orientation–it appears that the substitution of homologous exons has the effect of making the ligand binding pocket smaller for the 2HQQ isoform.
(PDF)

S13 Fig The superimposed structures of two isoforms of *MAPK14*. The two homologous exons are shown overlapping in orange (PDB structure: 1R39) and aquamarine (PDB structure: 3OHT) with the side chains represented as sticks. The main difference between the two homologous isoforms seems to be that the helices on the top right of the structure are displaced, though this could be in part because the 3OHT structure was resolved for salmon rather than human.
(PDF)

S14 Fig The superimposed structures of two isoforms of *PKM*. The two homologous exons are shown overlapping in yellow (PDB structure: 3SRF) and blue (PDB structure: 3SRD) with the side chains represented as sticks. The two structures are highly similar, though the changes in side chain and side chain orientation will produce subtle differences in function between the two isoforms.
(PDF)

S15 Fig The superimposed structures of two isoforms of *SNAP25*. The two homologous exons are shown overlapping in orange (PDB structure: 1JTH) and aquamarine (PDB structure: 3SFC) with the side chains represented as sticks. The two structures form coiled coils in conjunction with other proteins (not shown for clarity) and are highly similar, though again the changes in side chain and side chain orientation will produce subtle differences in function between the two isoforms.
(PDF)

S16 Fig The superimposed structures of two isoforms of *CDKN2A*. On the left, in orange, the PDB structure of 1D9S, on the right, in light blue, the structure of 1HN3. The structure of 1HN3 was resolved for mouse. The two isoforms are generated from overlapping exons in distinct frames, so there is no homology here. This is the only pair of structures that we find in the PDB for isoforms that were not generated from homologous exons.
(PDF)

S17 Fig The Pfam domain broken in the splice variants of *ATP2B1* and *ATP2B4*. A. Seed alignment for Pfam domain PF12424 (Plasma membrane calcium transporter ATPase C terminal). B. Structure resolved for this domain, from PDB structure 2KNE, chain B, which is a single helix, bound to calmodulin. C. The alignment between the two homologous isoforms of *ATP2B1* identified in the experiments. The blue and red arrows show where the Pfam seed alignment begins (blue) and where the conserved section of the Pfam domain ends (red). The Pfam domain is highly conserved over the first part of the alignment (the part of the alignment that coincides with the crystalised helix structure), but has little conservation at the C-terminal end. The (distant) homology detected for the *ATP2B1* isoforms (and all other members of this family) is clear up to the residue indicated with the red arrow, but it not conserved after and therefore the splice event would break the domain. However, the evidence from the PDB structure (and the lack of conservation in the Pfam seed alignment itself) suggests that the Pfam alignment should not be extended beyond the red arrow. In fact the residues before the first N-terminal residue of PF12424 are just as conserved as the residues between the red and blue arrows, suggesting that perhaps the plasma membrane calcium transporter ATPase C terminal domain should also be extended towards the N-terminal end.
(PDF)
