# Supplementary material for: Alternatively Spliced Homologous Exons Have Ancient Origins and Are Highly Expressed at the Protein Level
Source: PLoS Comput Biol. 2015 Jun 10;11(6):e1004325. doi: 10.1371/journal.pcbi.1004325 (PMC4465641; doi:10.1371/journal.pcbi.1004325)
Supplement: S1 Table — (PDF) [file pcbi.1004325.s001.pdf]

Data Set	Ezkurdia	Geiger	Kim	Munoz	Nagaraj	NIST	PeptAtlas	Wilhelm	Eight	GENCODE20
**PubMed ID**	**22446687**	**22278370**	**24870542**	**22108792**	**22068331**	**-**	**24939129**	**24870543**		
**Cell types**	Mixture	11 cell lines	30 tissues	Stem cells	HeLa cells	Mixture	Mixture	35 tissues/fluids (1)		
**Replicates**	Mixture	Three	Yes	No	No	Mixture	Mixture	No		
**SearchDB**	GENCODE	IPI	RefSeq	IPI	IPI	SwissProt	Ensembl (2)	UniProt	-	-
**Engine**	X!Tandem	Andromeda	Sequest/Mascot	Mascot	Andromeda	Multiple	Various	Andromeda	-	-
**Peptides**	59,475	68,071	122,781	53,028	80,074	123,416	110,269	105,492	149,612	693,324 (3)
**All genes**	9,586	8,756	11,464	7,043	8,049	10,988	10,112	7,971	12,716	19,906 (4)
**AS events**	154	104	192	105	109	166	208	125	280	45,346
**AS peptides**	134	189	430	146	266	444	400	299	562	77,724 (3)
**AS per gene**	0.016	0.012	0.017	0.015	0.014	0.015	0.021	0.016	0.022	2.278
**% AS peptides**	0.225	0.278	0.350	0.275	0.332	0.360	0.363	0.283	0.376	11.210

Table S1. Breakdown of the data from the individual MS analyses.
The identifier of each data set is from author first name or database name. “**PubMed ID**” refers to the article where the peptides came from; “**Cell types**” the types of tissues or cells used; “**Replicates**” shows the numbers of replicates of each tissue/cell where replicates were carried out; “**SearchDB**” is the database used in the mapping process; “**Engine**” is the software used to map the peptides; “**Peptides**” are the numbers of peptides after the filtering process; “**All genes**” shows the number of genes identified by at least a single filtered peptide; “**AS events**” is the number of splice events (identified by a single peptide) from each data set. “**AS peptides**” shows the number of peptides that map uniquely to alternative proteins in each data set; “**AS per gene**” is the number of alternative splice events identified per gene in each set and **“% AS peptides**” is the percentage of all identified peptides that map alternative isoforms.

Alternative isoforms are determined using APPRIS, as described in the main article. The data used in the analysis comes from a combination of the eight studies/databases and is shown here in the column “**Eight**”. For the “**GENCODE20**” column the data comes from the whole genome, so the “AS events” are the number of alternative splice events annotated in the whole genome, the “**AS per gene**” is the number of annotated alternative splice events per gene, and the **“% AS peptides**” is the percentage of tryptic peptides in the whole genome that map exclusively to alternative isoforms. The table allows us to make a comparison between the number of identified AS events per gene and the number of annotated splice events per gene (we identify less than 1% of the AS events) and between the percentages of identified and annotated peptides that map to alternative isoforms (we identify proportionally thirty times fewer alternative peptides than are annotated in the genome).

Notes:

(1) The Wilhelm et al. 2014, Nature paper reports differing numbers of tissues, we only had access to the results for 35 tissue/fluid samples
(2) In fact PeptideAtlas searches against compound databases, here the database was Ensembl/SwissProt/IPI combined
(3) These results are calculated for tryptic peptides only
(4) GENCODE20 coding genes minus the flagged read-through transcripts
