# Supplementary material for: Alternatively Spliced Homologous Exons Have Ancient Origins and Are Highly Expressed at the Protein Level
Source: PLoS Comput Biol. 2015 Jun 10;11(6):e1004325. doi: 10.1371/journal.pcbi.1004325 (PMC4465641; doi:10.1371/journal.pcbi.1004325)
Supplement: S1 Fig — A. Multiple alignment of the N-terminal region of seven of the isoforms of the UGT1A cluster that were identified in the analysis. The peptides we detected in the eight data sets are mapped onto the alignment in colour. Although all peptides discriminate between some isoforms (even though they do not fall in the splice junction) red peptides discriminate between all splice isoforms, blue peptides only discriminate some of the isoforms. B. The UGT1A cluster exons. (PDF) [file pcbi.1004325.s004.pdf]

A

UGT1A10	MARAGWTSPVP-LCVCLLLTCG---FA--EAGKLLVVPMDGSHWFTMQSVVEKLILRGHE
UGT1A9	MACTGWTSPLP-LCVCLLLTCG---FA--EAGKLLVVPMDGSHWFTMRSVVEKLILRGHE
UGT1A7	MARTGWTSPIP-LCVSLLLTCG---FA--EAGKLLVVPMDGSHWFTMQSVVEKLILRGHE
UGT1A6	MAC--LLRSFQRISAGVFFLAL---WGMVVGDKLLVVPQDGSHWLSMKDIVEVLSDRGHE
UGT1A4	MAR-GLQVPLPRLATGLLLLLSVQPWA--ESGKVLVVPTDGSPWLSMREALRELHARGHQ
UGT1A3	MAT-GLQVPLPWLATGLLLLLSVQPWA--ESGKVLVVPIDGSHWLSMREVLRELHARGHQ
UGT1A8	MAV-ESQGGRP-LVLGLLLCVLGPVVS--HAGKILLIPVDGSHWLSMLGAIQQLQQRGHE
UGT1A1	MARTGWTSPIP-LCVSLLLTCG---FA--EAGKLLVVPMDGSHWFTMQSVVEKLILRGHE
UGT1A10	VVVVMPEVSWQLERSLNCTVKTYSTSYTLEDQNREFMVFAHAQWKAQ-AQSIFSLLMSSS
UGT1A9	VVVVMPEVSWQLGRSLNCTVKTYSTSYTLEDLDREFKAFAHAQWKAQ-VRSIYSLLMGSY
UGT1A7	VVVVMPEVSWQLGRSLNCTVKTYSTSYTLEDQDREFMVFADARWTAP-LRSAFSLLTSSS
UGT1A6	IVVVVPEVNLLLKESKYYTRKIYPVPYDQEELKNRYQSFGNNHFAERSFLTAPQTEYRNN
UGT1A4	AVVLTPEVNMHIKEEKFFTLTAYAVPWTQKEFDRVTLGYTQGFFETEHLLKRYSRSMAIM
UGT1A3	AVVLTPEVNMHIKEENFFTLTTYAISWTQDEFDRHVLGHTQLYFETEHFLKKFFRSMAML
UGT1A8	IVVLAPDASLYIRDGAFYTLKTYPVPFQREDVKESFVSLGHNVFENDSFLQRVIKTYKKI
UGT1A1	VVVVMPEVSWQLGKSLNCTVKTYSTSYTLEDLDREFMDFADAQWKAQ-VRSLFSLFLSSS

B

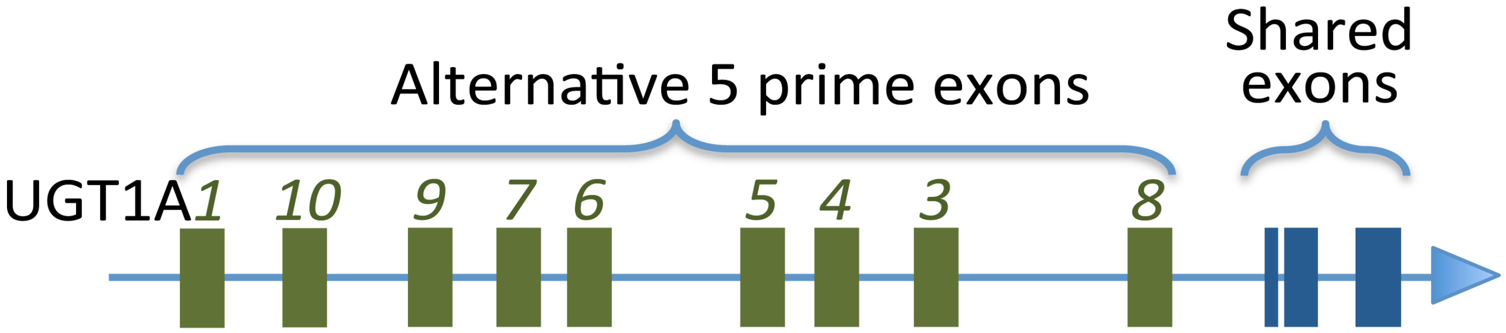

Figure S1. Splice event specific peptides for the UGT1A cluster
A. Multiple alignment of the N-terminal region of seven of the isoforms of the UGT1A cluster that were identified in the analysis. The peptides we detected in the eight data sets are mapped onto the alignment in colour. Although all peptides discriminate between some isoforms (even though they do not fall in the splice junction) red peptides discriminate between all splice isoforms, blue peptides only discriminate some of the isoforms. B. The UGT1A cluster exons.
