# Supplementary material for: Alternatively Spliced Homologous Exons Have Ancient Origins and Are Highly Expressed at the Protein Level
Source: PLoS Comput Biol. 2015 Jun 10;11(6):e1004325. doi: 10.1371/journal.pcbi.1004325 (PMC4465641; doi:10.1371/journal.pcbi.1004325)
Supplement: S2 Fig — A portion of the gene model of HNRPNC from the Ensembl web page. The two isoforms 002 and 007 differ by the insertion/deletion of 13 amino acid residues at the N-terminal (5’ end) of the protein. Here the second exon is missing in variant 007. (PDF) [file pcbi.1004325.s005.pdf]

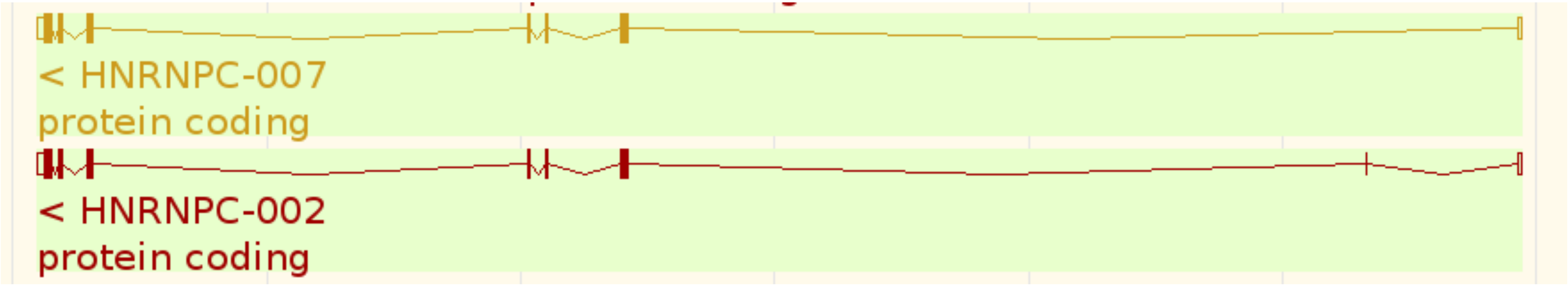

Figure S2. The indel detected for gene *HNRNPC*.
A portion of the gene model of HNRPNC from the Ensembl web page. The two isoforms 002 and 007 differ by the insertion/deletion of 13 amino acid residues at the N-terminal (5' end) of the protein. Here the second exon is missing in variant 007.
