# Supplementary material for: Alternatively Spliced Homologous Exons Have Ancient Origins and Are Highly Expressed at the Protein Level
Source: PLoS Comput Biol. 2015 Jun 10;11(6):e1004325. doi: 10.1371/journal.pcbi.1004325 (PMC4465641; doi:10.1371/journal.pcbi.1004325)
Supplement: S4 Fig — A section of the TMPO gene model from the Ensembl web pages. Here the large 3’ exon in variant 001 is replaced by five smaller coding exons in variants 002 and 014. The substitution is not homologous. TMPO was one of the genes with the clearest evidence of protein level alternative splicing; one of the alternative exons was derived from a transposon [84]. (PDF) [file pcbi.1004325.s007.pdf]

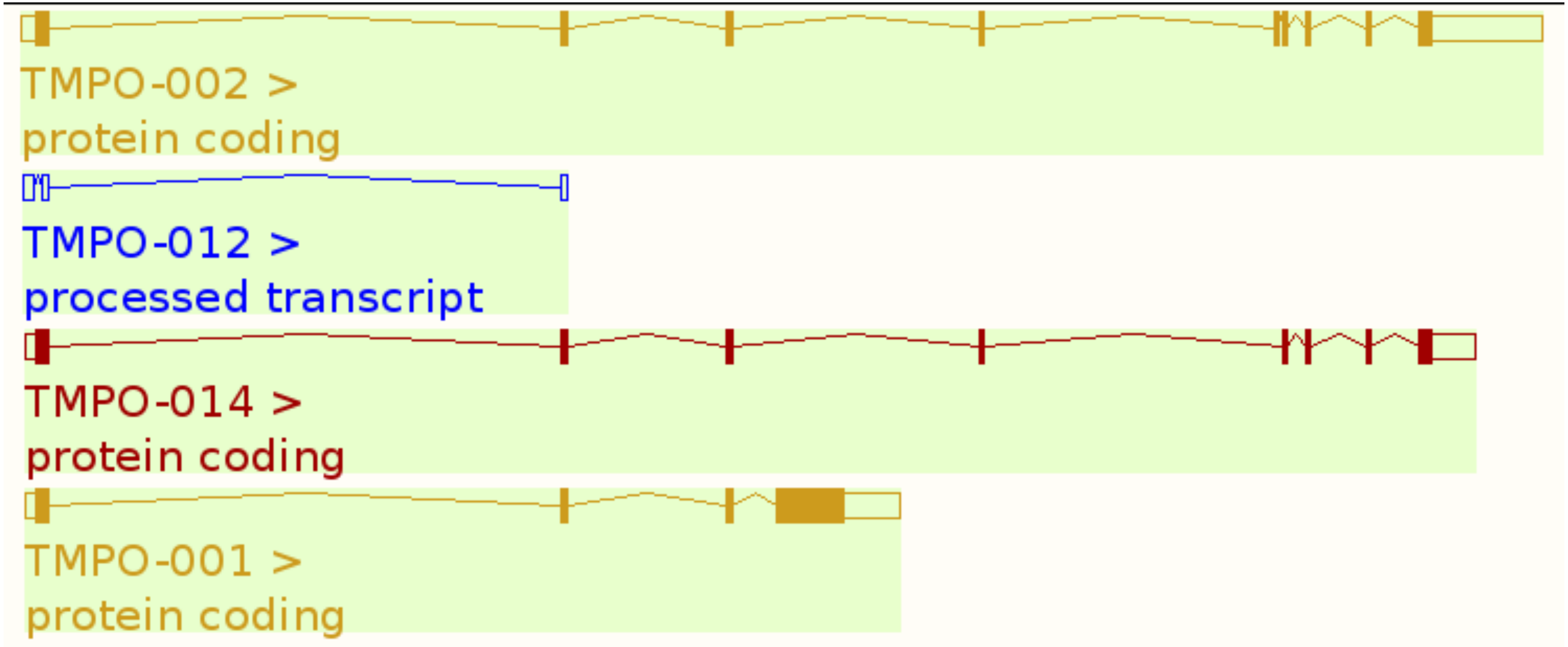

Figure S4. C-terminal substitution for *TMPO*.
A section of the *TMPO* gene model from the Ensembl web pages. Here the large 3' exon in variant 001 is replaced by five smaller coding exons in variants 002 and 014. The substitution is not homologous. *TMPO* was one of the genes with the clearest evidence of protein level alternative splicing; one of the alternative exons was derived from a transposon (**83**).
