# Supplementary material for: Alternatively Spliced Homologous Exons Have Ancient Origins and Are Highly Expressed at the Protein Level
Source: PLoS Comput Biol. 2015 Jun 10;11(6):e1004325. doi: 10.1371/journal.pcbi.1004325 (PMC4465641; doi:10.1371/journal.pcbi.1004325)
Supplement: S5 Fig — A section of the NEBL gene model from the Ensembl web pages showing the two most important isoforms, nebulette (004) and LIM-nebulette (003). Most if the 5’ exons of nebulette are replaced by just four exons in LIM-nebulette, though LIM-nebulatte does add a LIM domain. (PDF) [file pcbi.1004325.s008.pdf]

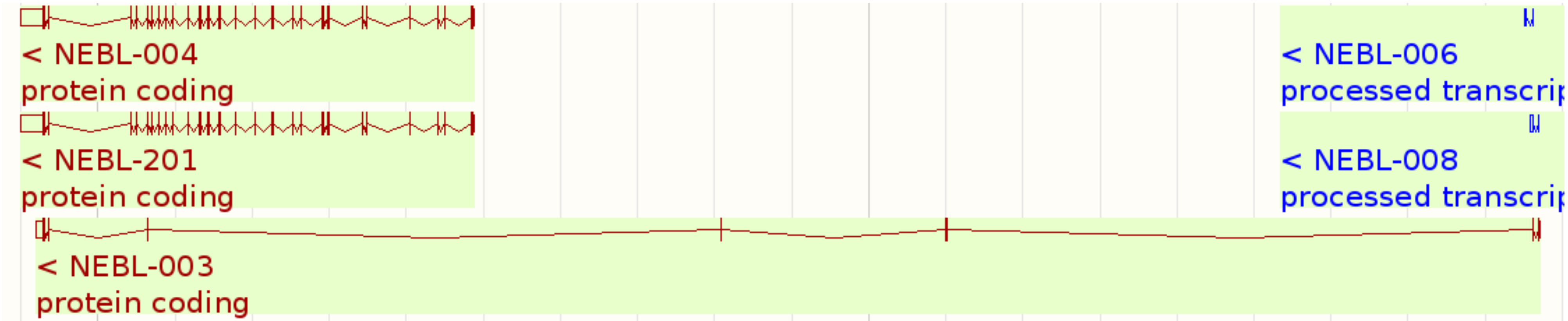

Figure S5. N-terminal substitution for *NEBL*.
A section of the *NEBL* gene model from the Ensembl web pages showing the two most important isoforms, nebulette (004) and LIM-nebulette (003). Most if the 5’ exons of nebulette are replaced by just four exons in LIM-nebulette, though LIM-nebulatte does add a LIM domain.
