# Supplementary material for: Alternatively Spliced Homologous Exons Have Ancient Origins and Are Highly Expressed at the Protein Level
Source: PLoS Comput Biol. 2015 Jun 10;11(6):e1004325. doi: 10.1371/journal.pcbi.1004325 (PMC4465641; doi:10.1371/journal.pcbi.1004325)
Supplement: S6 Fig — A section of the pairwise alignment between two KIF23 isoforms. The internal exon substitution results in a swap 100 residues for just six residues. There is no homology. These substitutions were very rare in the proteomics experiments. (PDF) [file pcbi.1004325.s009.pdf]

```
ENST00000395392 RNLQQELETQNQKLQRQFSDKRRLEARLQGMVTETTMKWEKECERRVAAKQLEMQNKLWV
ENST00000352331 RNLQQELETQNQKLQRQFSDKRRLEARLQGMVTETTMKWEKECERRVAAKQLEMQNKLWV

ENST00000395392 KDEKLKQLKAIVTEPKTEKPERPSRERDREKVTQRSVSPSPVPLSSNYIAQISNGQQLMS
ENST00000352331 KDEKLKQLKAIVTEPKTEKPERPSRERDREKVTQRSVSPSPVP-----------------

ENST00000395392 QPQLHRRSNSCSSISVASCISEWEQKIPTYNTPLKVTSIARRRQQEPGQSKTCIVSDRRR
ENST00000352331 ------------------------------------------------------------

ENST00000395392 GMYWTEGREVVPTFRNEIEIEEDHCG-------RNAPPIRLRHRRSRSAGDRWVDHKPAS
ENST00000352331 ---------------------------LLFQPDQNAPPIRLRHRRSRSAGDRWVDHKPAS

ENST00000395392 NMQTETVMQPHVPHAITVSVANEKALAKCEKYMLTHQELASDGEIETKLIKGDIYKTRGG
ENST00000352331 NMQTETVMQPHVPHAITVSVANEKALAKCEKYMLTHQELASDGEIETKLIKGDIYKTRGG
```

Figure S6. *KIF23* Internal substitution.
A section of the pairwise alignment between two *KIF23* isoforms. The internal exon substitution results in a swap 100 residues for just six residues. There is no homology. These substitutions were very rare in the proteomics experiments.
