# Supplementary material for: Alternatively Spliced Homologous Exons Have Ancient Origins and Are Highly Expressed at the Protein Level
Source: PLoS Comput Biol. 2015 Jun 10;11(6):e1004325. doi: 10.1371/journal.pcbi.1004325 (PMC4465641; doi:10.1371/journal.pcbi.1004325)
Supplement: S7 Fig — The Ensembl gene model for the MICAL3 gene. Isoforms 001 and 002 would be translated from one set of overlapping exons while 011 and 017 would be translated from a different set of non-overlapping exons. (PDF) [file pcbi.1004325.s010.pdf]

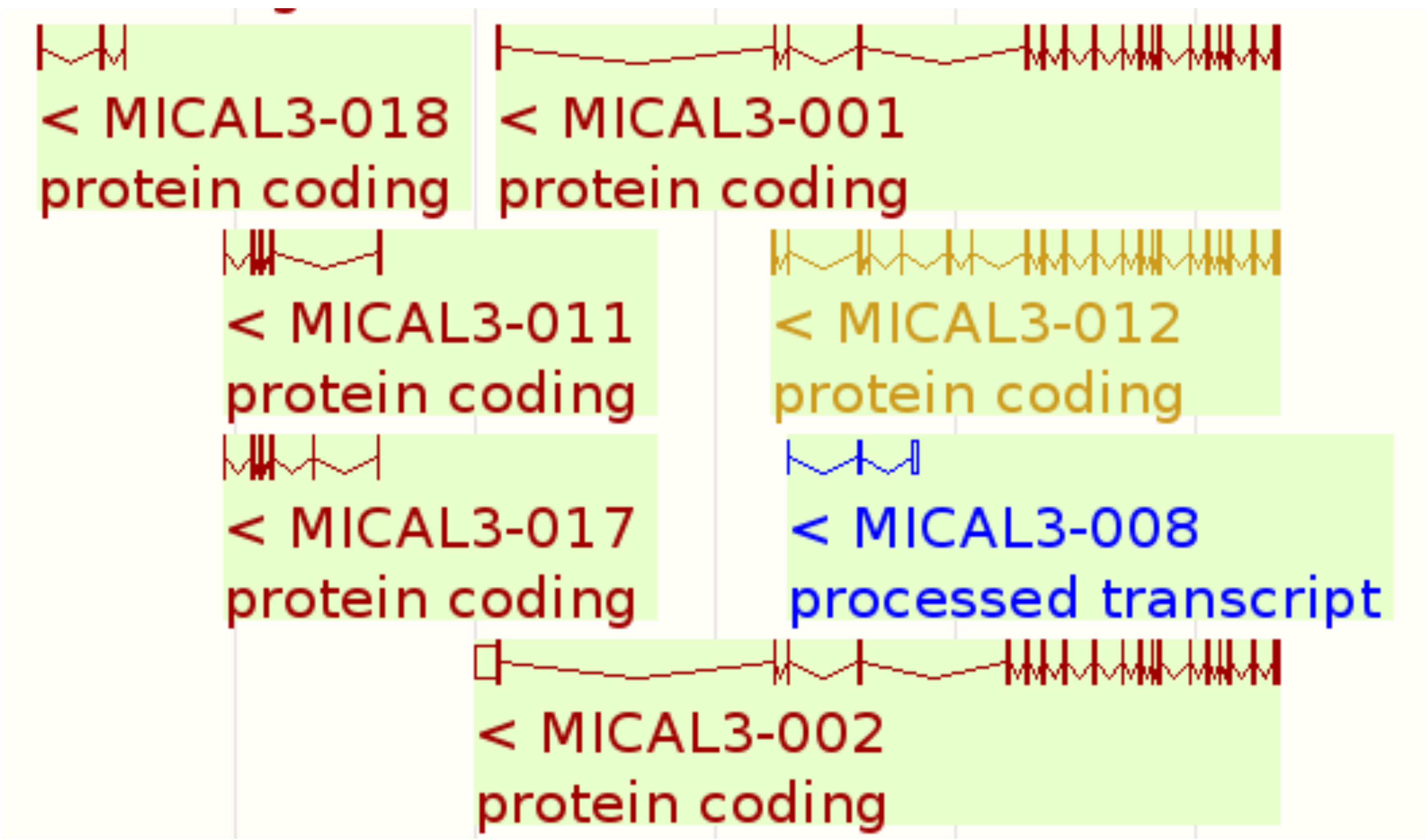

Figure S7. *MICAL3* gene would give rise to two unique proteins.
The Ensembl gene model for the *MICAL3* gene. Isoforms 001 and 002 would be translated from one set of overlapping exons while 011 and 017 would be translated from a different set of non-overlapping exons.
