# Supplementary material for: Alternatively Spliced Homologous Exons Have Ancient Origins and Are Highly Expressed at the Protein Level
Source: PLoS Comput Biol. 2015 Jun 10;11(6):e1004325. doi: 10.1371/journal.pcbi.1004325 (PMC4465641; doi:10.1371/journal.pcbi.1004325)
Supplement: S8 Fig — This histogram shows the peptide abundance for all genes with at least 1 peptide (in green) and the genes for which we detected evidence of alternative splicing (AS genes, red). As expected, alternative splicing detection is related to peptide abundance in proteomics experiments (which here is the measure of levels of protein expression); the more peptides we detect for a gene, the more likely we are to detect alternative splicing. (PDF) [file pcbi.1004325.s011.pdf]

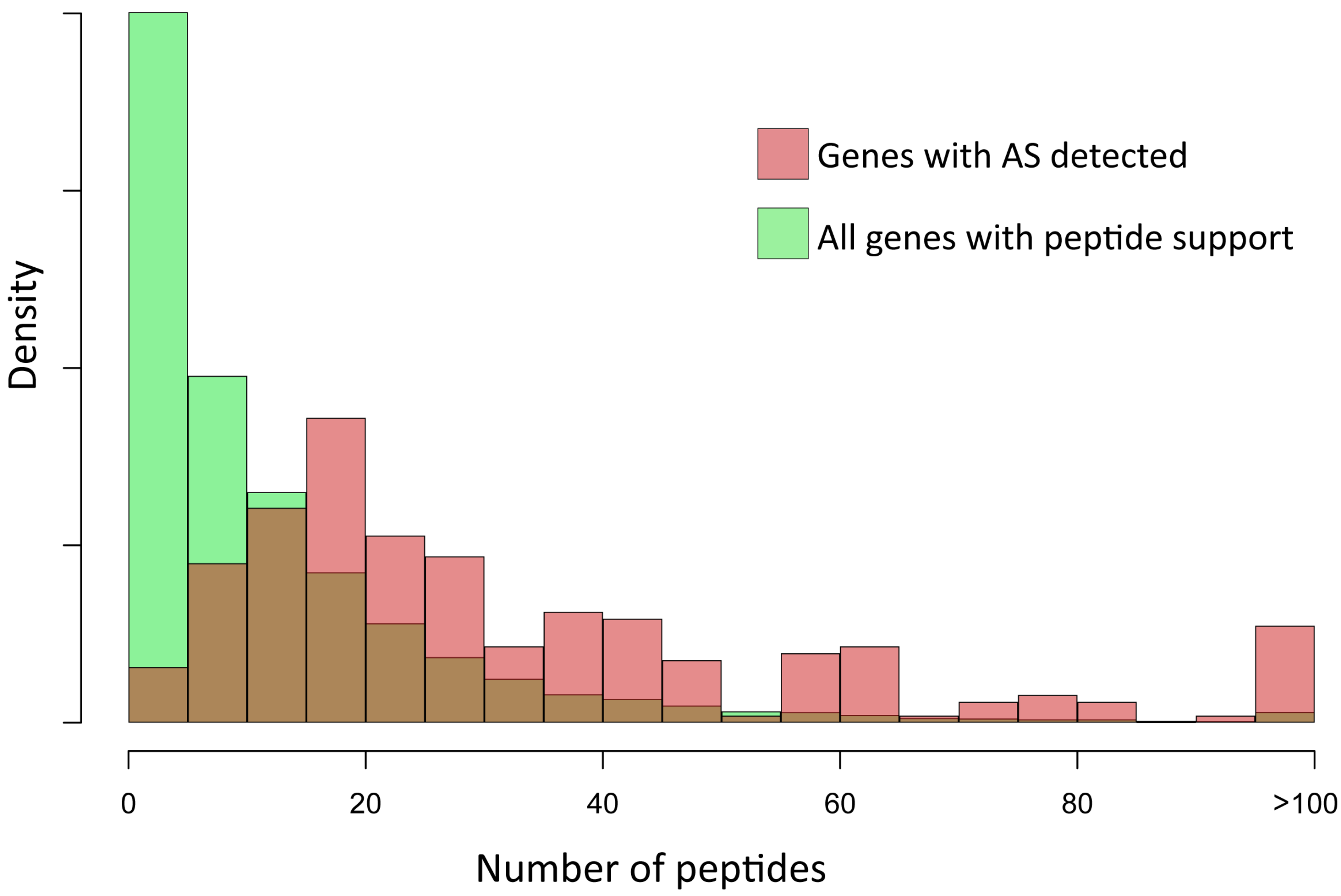

Figure S8. Histogram of peptides detected for AS and all genes
This histogram shows the peptide abundance for all genes with at least 1 peptide (in green) and the genes for which we detected evidence of alternative splicing (AS genes, red). As expected, alternative splicing detection is related to peptide abundance in proteomics experiments (which here is the measure of levels of protein expression); the more peptides we detect for a gene, the more likely we are to detect alternative splicing.
