# Supplementary material for: Alternatively Spliced Homologous Exons Have Ancient Origins and Are Highly Expressed at the Protein Level
Source: PLoS Comput Biol. 2015 Jun 10;11(6):e1004325. doi: 10.1371/journal.pcbi.1004325 (PMC4465641; doi:10.1371/journal.pcbi.1004325)
Supplement: S9 Fig — The percentage of each type of splice event detected in each of the eight peptide data sets. The types of splicing events are described in the main paper. Splice events could be identified in the individual data sets with just a single peptide for each side of the event. This is the figure version of supplementary S3 Table. (PDF) [file pcbi.1004325.s012.pdf]

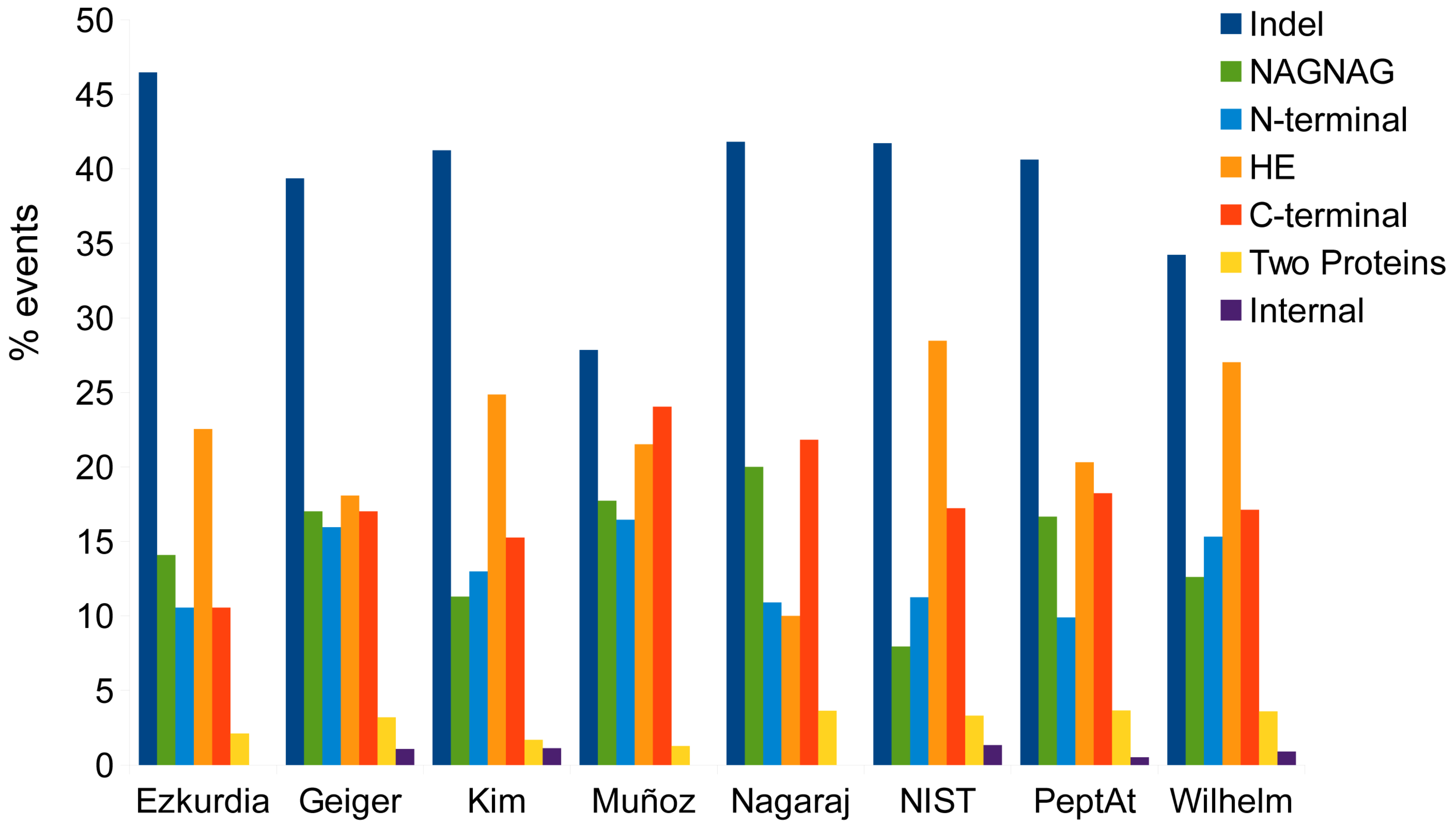

Figure S9. Types of splicing events found in each experiment
The percentage of each type of splice event detected in each of the eight peptide data sets. The types of splicing events are described in the main paper. Splice events could be identified in the individual data sets with just a single peptide for each side of the event. This is the figure version of supplementary table S2.
