# Supplementary material for: Alternatively Spliced Homologous Exons Have Ancient Origins and Are Highly Expressed at the Protein Level
Source: PLoS Comput Biol. 2015 Jun 10;11(6):e1004325. doi: 10.1371/journal.pcbi.1004325 (PMC4465641; doi:10.1371/journal.pcbi.1004325)
Supplement: S10 Fig — The percentage of each type of splice event for those splice events detected across multiple experiments. Splice events in the first column are those events that were detected in all eight experiments, the second column the events were detected in seven of the eight individual experiments, the third column in six individual experiments, and so on. The types of splicing events are described in the main paper. Splice events could be identified in the individual data sets with just a single peptide for each side of the event. (PDF) [file pcbi.1004325.s013.pdf]

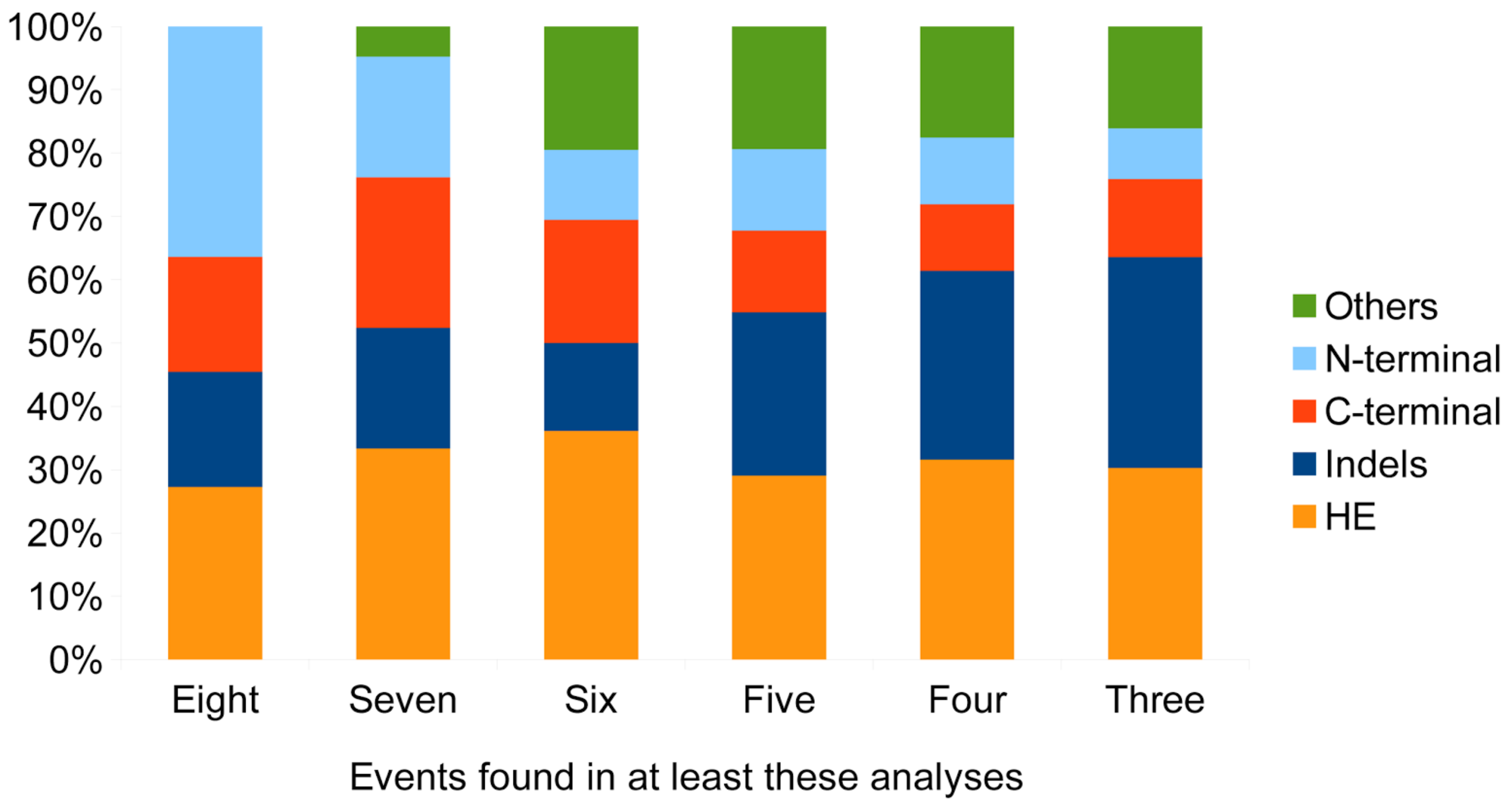

Figure S10. Splicing events found in multiple experiments
The percentage of each type of splice event for those splice events detected across multiple experiments. Splice events in the first column are those events that were detected in all eight experiments, the second column the events were detected in seven of the eight individual experiments, the third column in six individual experiments, and so on. The types of splicing events are described in the main paper. Splice events could be identified in the individual data sets with just a single peptide for each side of the event.
