# Supplementary material for: Alternatively Spliced Homologous Exons Have Ancient Origins and Are Highly Expressed at the Protein Level
Source: PLoS Comput Biol. 2015 Jun 10;11(6):e1004325. doi: 10.1371/journal.pcbi.1004325 (PMC4465641; doi:10.1371/journal.pcbi.1004325)
Supplement: S11 Fig — The two homologous exons are shown overlapping in orange (PDB structure: 4UUD) and aquamarine (PDB structure: 3ZVR) with the side chains represented as sticks. The two structures have relatively large differences for structures produced from homologous exons. In part this will be because the one of the dynamin isoforms (3ZVR) was resolved for rat rather than human, and in part will be because helical structures are generally more flexible than beta-sheet based structures. (PDF) [file pcbi.1004325.s014.pdf]

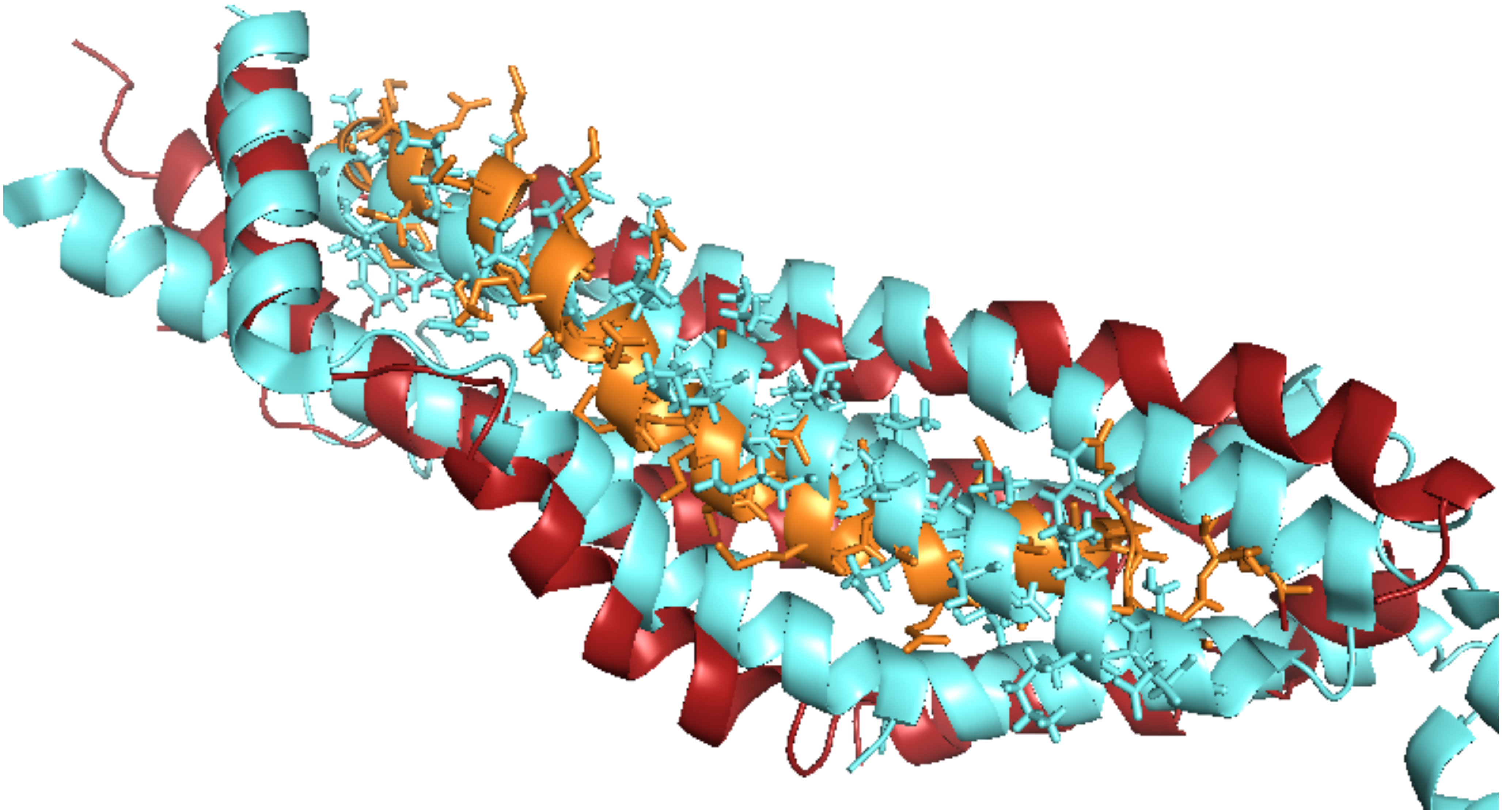

**Figure S11. The superimposed structures of two isoforms of *DNM1*.**
The two homologous exons are shown overlapping in orange (PDB structure: 4UUD) and aquamarine (PDB structure: 3ZVR) with the side chains represented as sticks. The two structures have relatively large differences for structures produced from homologous exons. In part this will be because the one of the dynamin isoforms (3ZVR) was resolved for rat rather than human, and in part will be because helical structures are generally more flexible than beta-sheet based structures.
