# Supplementary material for: Alternatively Spliced Homologous Exons Have Ancient Origins and Are Highly Expressed at the Protein Level
Source: PLoS Comput Biol. 2015 Jun 10;11(6):e1004325. doi: 10.1371/journal.pcbi.1004325 (PMC4465641; doi:10.1371/journal.pcbi.1004325)
Supplement: S12 Fig — The two homologous exons are shown overlapping in orange (PDB structure: 3B3L) and aquamarine (PDB structure: 2HQQ) with the side chains represented as sticks. Interestingly the structural superposition allows us to make a hypothesis for a subtle change in function for between the two ketohexokinase isoforms. While the catalytic residues identified by firestar superimpose well, the ligand binding residues (shown in yellow) in the region of the homologous exon have a different orientation–it appears that the substitution of homologous exons has the effect of making the ligand binding pocket smaller for the 2HQQ isoform. (PDF) [file pcbi.1004325.s015.pdf]

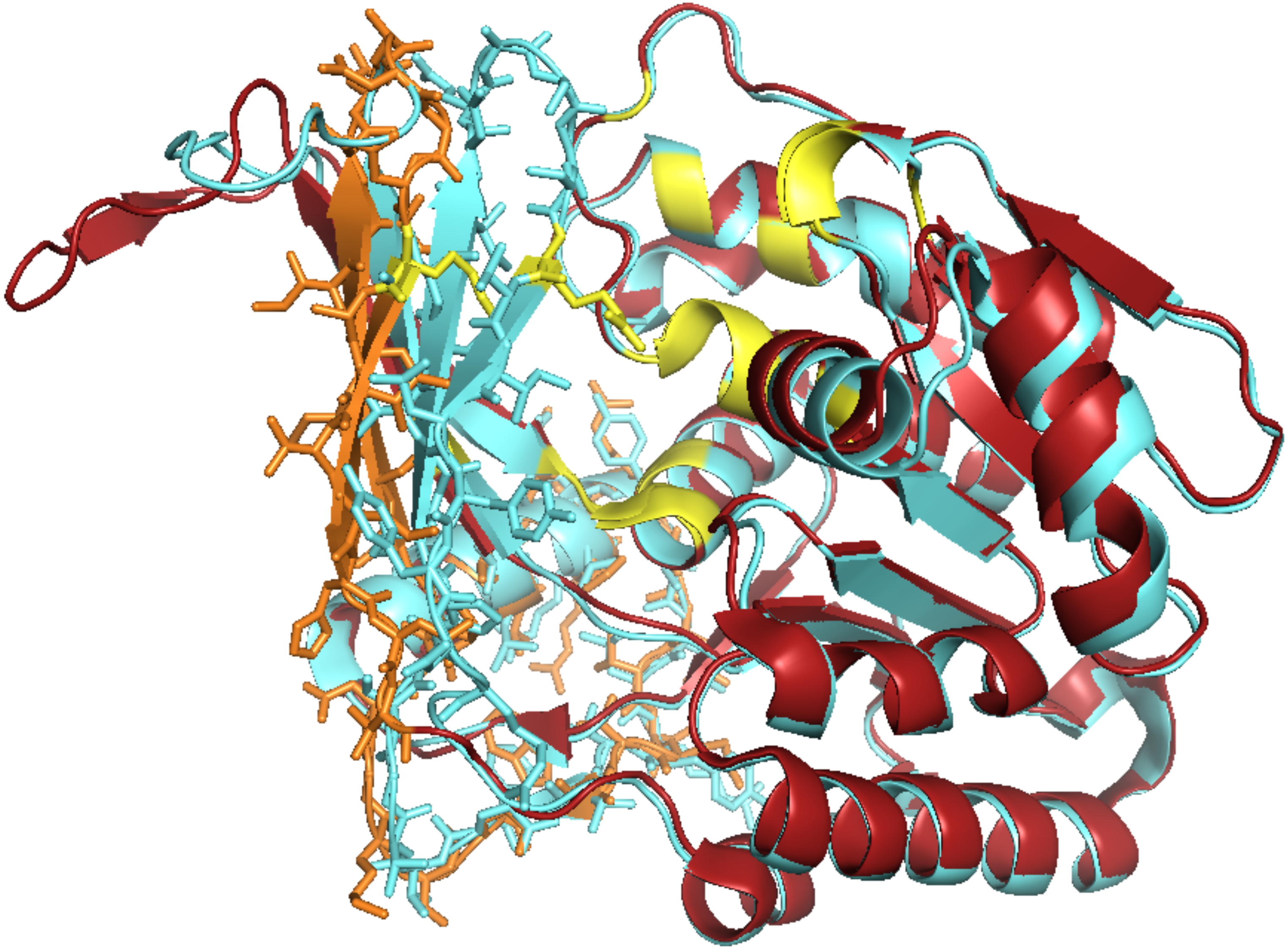

Figure S12. The superimposed structures of two isoforms of *KHK*.
The two homologous exons are shown overlapping in orange (PDB structure: 3B3L) and aquamarine (PDB structure: 2HQQ) with the side chains represented as sticks. Interestingly the structural superposition allows us to make a hypothesis for a subtle change in function for between the two ketohexokinase isoforms. While the catalytic residues identified by *firestar* (**84**) superimpose well, the ligand binding residues (shown in yellow) in the region of the homologous exon have a different orientation – it appears that the substitution of homologous exons has the effect of making the ligand binding pocket smaller for the 2HQQ isoform.
