# Supplementary material for: Alternatively Spliced Homologous Exons Have Ancient Origins and Are Highly Expressed at the Protein Level
Source: PLoS Comput Biol. 2015 Jun 10;11(6):e1004325. doi: 10.1371/journal.pcbi.1004325 (PMC4465641; doi:10.1371/journal.pcbi.1004325)
Supplement: S13 Fig — The two homologous exons are shown overlapping in orange (PDB structure: 1R39) and aquamarine (PDB structure: 3OHT) with the side chains represented as sticks. The main difference between the two homologous isoforms seems to be that the helices on the top right of the structure are displaced, though this could be in part because the 3OHT structure was resolved for salmon rather than human. (PDF) [file pcbi.1004325.s016.pdf]

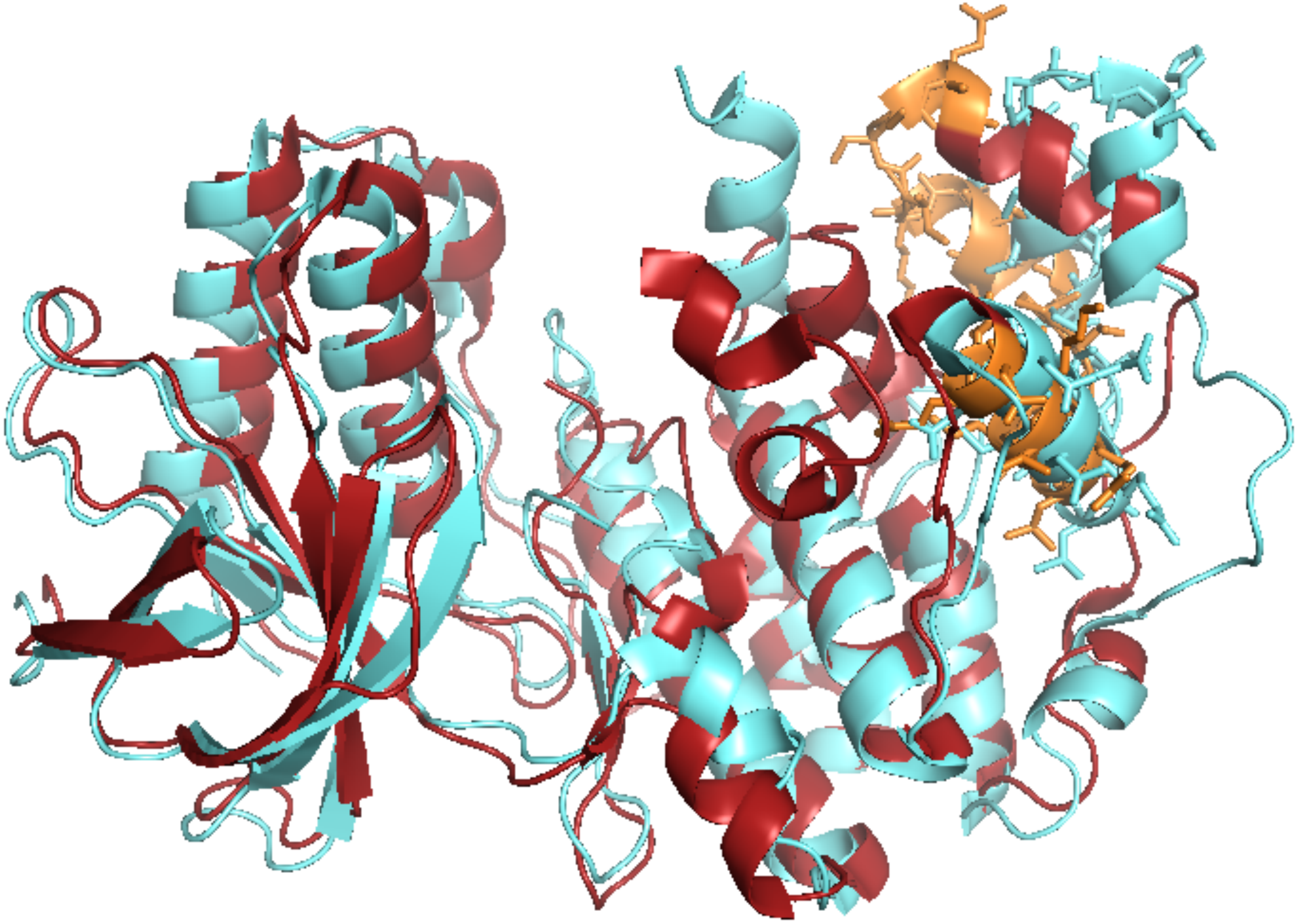

Figure S13. The superimposed structures of two isoforms of *MAPK14*.
The two homologous exons are shown overlapping in orange (PDB structure: 1R39) and aquamarine (PDB structure: 3OHT) with the side chains represented as sticks. The main difference between the two homologous isoforms seems to be that the helices on the top right of the structure are displaced, though this could be in part because the 3OHT structure was resolved for salmon rather than human.
