# Supplementary material for: Alternatively Spliced Homologous Exons Have Ancient Origins and Are Highly Expressed at the Protein Level
Source: PLoS Comput Biol. 2015 Jun 10;11(6):e1004325. doi: 10.1371/journal.pcbi.1004325 (PMC4465641; doi:10.1371/journal.pcbi.1004325)
Supplement: S14 Fig — The two homologous exons are shown overlapping in yellow (PDB structure: 3SRF) and blue (PDB structure: 3SRD) with the side chains represented as sticks. The two structures are highly similar, though the changes in side chain and side chain orientation will produce subtle differences in function between the two isoforms. (PDF) [file pcbi.1004325.s017.pdf]

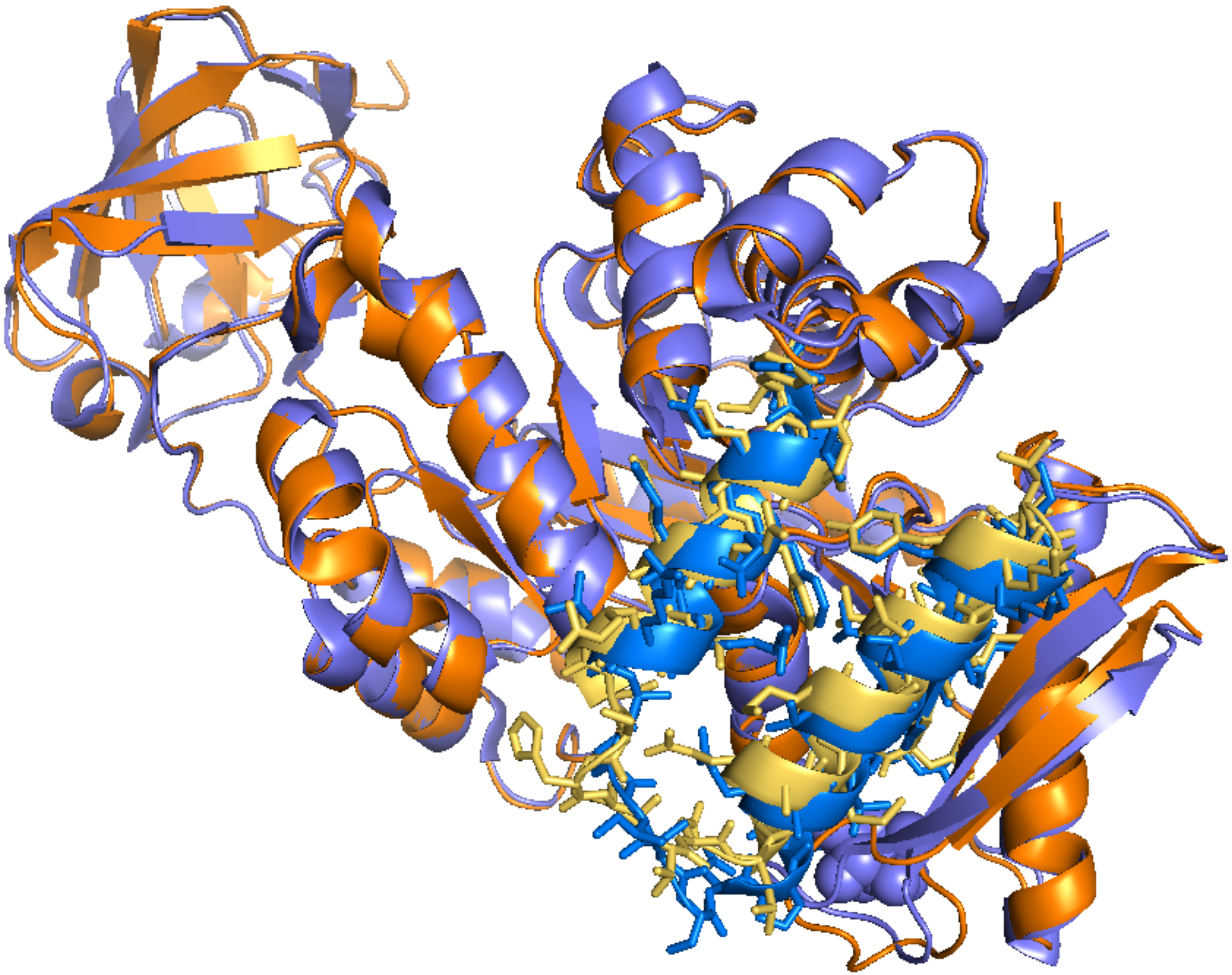

Figure S14. The superimposed structures of two isoforms of *PKM*.
The two homologous exons are shown overlapping in yellow (PDB structure: 3SRF) and blue (PDB structure: 3SRD) with the side chains represented as sticks. The two structures are highly similar, though the changes in side chain and side chain orientation will produce subtle differences in function between the two isoforms.
