# Supplementary material for: Alternatively Spliced Homologous Exons Have Ancient Origins and Are Highly Expressed at the Protein Level
Source: PLoS Comput Biol. 2015 Jun 10;11(6):e1004325. doi: 10.1371/journal.pcbi.1004325 (PMC4465641; doi:10.1371/journal.pcbi.1004325)
Supplement: S15 Fig — The two homologous exons are shown overlapping in orange (PDB structure: 1JTH) and aquamarine (PDB structure: 3SFC) with the side chains represented as sticks. The two structures form coiled coils in conjunction with other proteins (not shown for clarity) and are highly similar, though again the changes in side chain and side chain orientation will produce subtle differences in function between the two isoforms. (PDF) [file pcbi.1004325.s018.pdf]

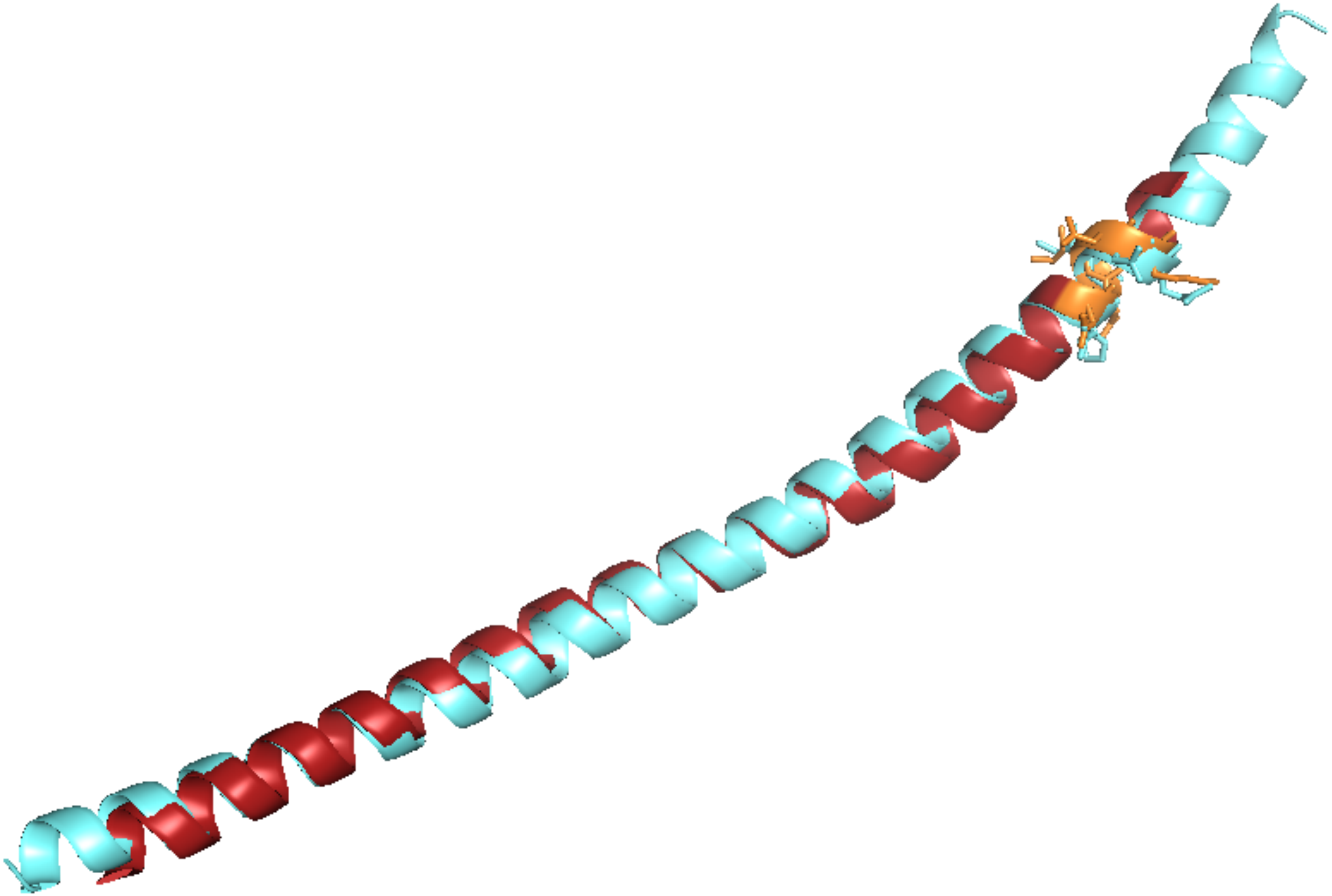

Figure S15. The superimposed structures of two isoforms of *SNAP25*.
The two homologous exons are shown overlapping in orange (PDB structure: 1JTH) and aquamarine (PDB structure: 3SFC) with the side chains represented as sticks. The two structures form coiled coils in conjunction with other proteins (not shown for clarity) and are highly similar, though again the changes in side chain and side chain orientation will produce subtle differences in function between the two isoforms.
