# Supplementary material for: Alternatively Spliced Homologous Exons Have Ancient Origins and Are Highly Expressed at the Protein Level
Source: PLoS Comput Biol. 2015 Jun 10;11(6):e1004325. doi: 10.1371/journal.pcbi.1004325 (PMC4465641; doi:10.1371/journal.pcbi.1004325)
Supplement: S16 Fig — On the left, in orange, the PDB structure of 1D9S, on the right, in light blue, the structure of 1HN3. The structure of 1HN3 was resolved for mouse. The two isoforms are generated from overlapping exons in distinct frames, so there is no homology here. This is the only pair of structures that we find in the PDB for isoforms that were not generated from homologous exons. (PDF) [file pcbi.1004325.s019.pdf]

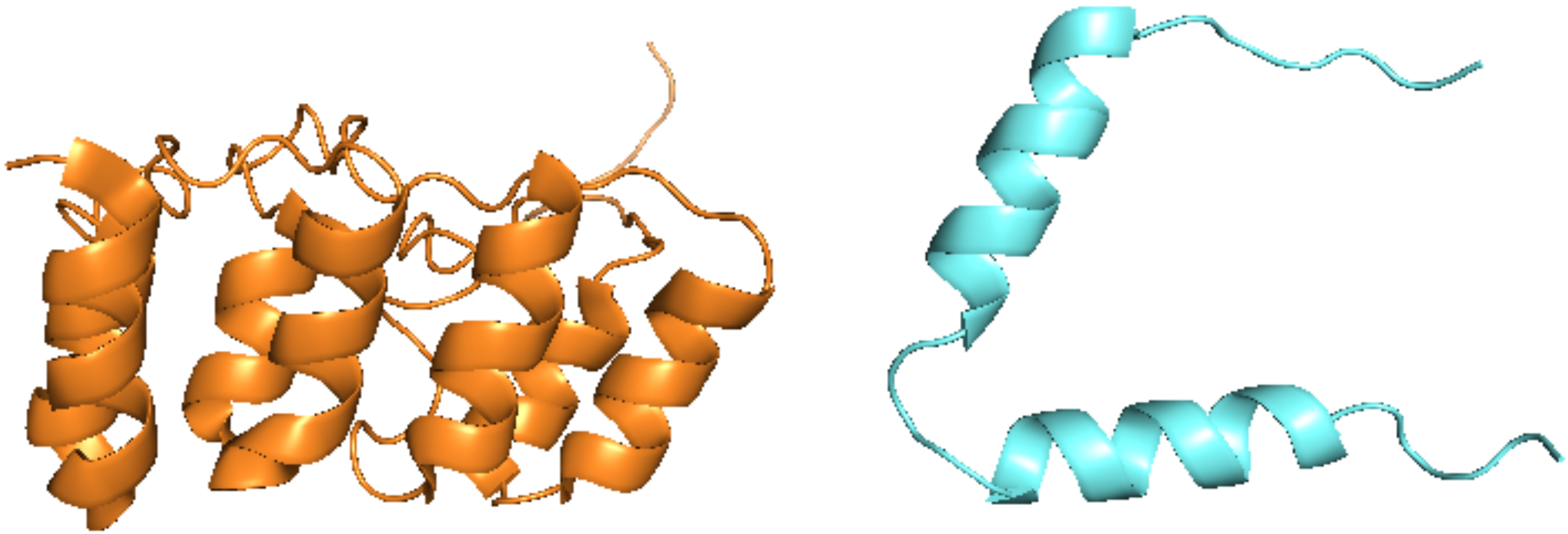

Figure S16. The superimposed structures of two isoforms of *CDKN2A*.
On the left, in orange, the PDB structure of 1D9S, on the right, in light blue, the structure of 1HN3. The structure of 1HN3 was resolved for mouse. The two isoforms are generated from overlapping exons in distinct frames, so there is no homology here. This is the only pair of structures that we find in the PDB for isoforms that were not generated from homologous exons.
