# Supplementary material for: Alternatively Spliced Homologous Exons Have Ancient Origins and Are Highly Expressed at the Protein Level
Source: PLoS Comput Biol. 2015 Jun 10;11(6):e1004325. doi: 10.1371/journal.pcbi.1004325 (PMC4465641; doi:10.1371/journal.pcbi.1004325)
Supplement: S3 Fig — In A, a section of the CALU gene model from the Ensembl web pages, with arrows showing the homologous exclusively spliced exons for variants 001 and 002. In fact there are three splice events shown in this image, the homologous substitution, an N-terminal extension, which comes from the extra exon at the 5’ end in variants 003 and 005, and which cannot be detected by proteomics data, and a C-terminal substitution that results from skipping the penultimate coding exon in 006 (a C-terminal substitution in protein terms since the frame of the last exon is changed by the exon skip event. In B part of the pairwise alignment between the protein sequences of variants 001 (ENST00000249364) and 002 (ENST00000449187) showing the similarity between the two protein sequences that results from the homologous splicing event. Homologous exon events can also occur with the 5’ and 3’ exons. (PDF) [file pcbi.1004325.s006.pdf]

A

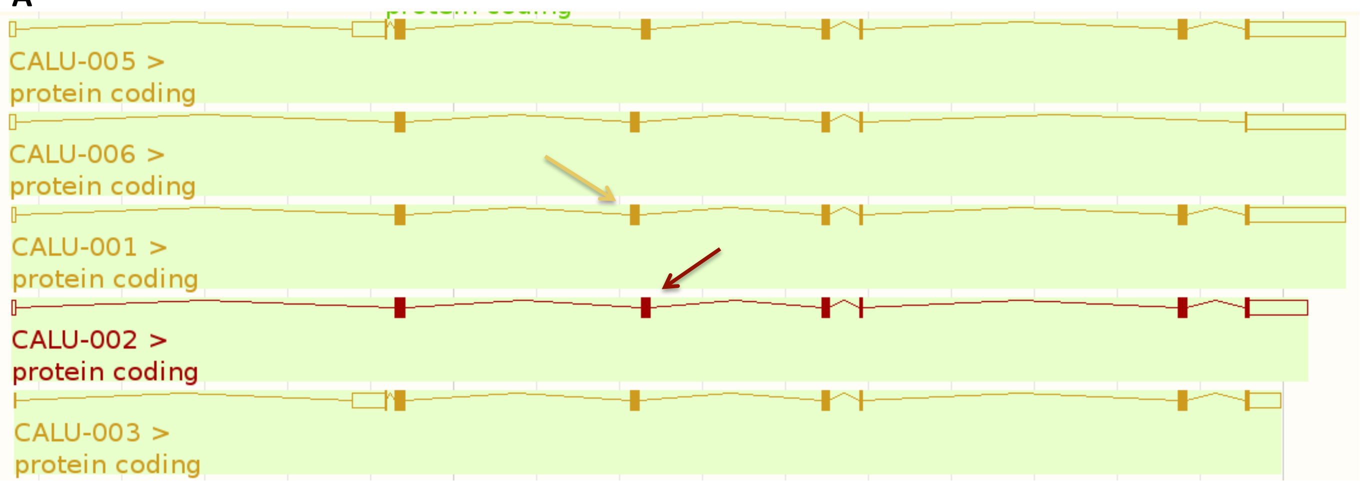

B

```
ENST00000249364        --------MDLRQFLMCLSLCTAFALSKPTEKKDRVHHEPQLSDKVHNDAQSFDYDHDAF
ENST00000449187        --------MDLRQFLMCLSLCTAFALSKPTEKKDRVHHEPQLSDKVHNDAQSFDYDHDAF
                               ****************************************************

ENST00000249364        LGAEEAKTFDQLTPEESKERLGKIVSKIDGDKDGFVTVDELKDWIKFAQKRWIYEDVERQ
ENST00000449187        LGAEEAKTFDQLTPEESKERLGMIVDKIDADKDGFVTEGELKSWIKHAQKKYIYDNVENQ
                       ********************** **.***.******* .***.***.***.:**::**.*

ENST00000249364        WKGHDLNEDGLVSWEEYKNATYGYVLDDPDPDDGFNYKQMMVRDERRFKMADKDGDLIAT
ENST00000449187        WQEFDMNQDGLISWDEYRNVTYGTYLDDPDPDDGFNYKQMMVRDERRFKMADKDGDLIAT
                       *: .*:*:***:**:**.*.***  ***********************************

ENST00000249364        KEEFTAFLHPEEYDYMKDIVVQETMEDIDKNADGFIDLEEYIGDMYSHDGNTDEPEWVKT
ENST00000449187        KEEFTAFLHPEEYDYMKDIVVQETMEDIDKNADGFIDLEEYIGDMYSHDGNTDEPEWVKT
                       ************************************************************
```

Figure S3. Mutually exclusive homologous exons for gene *CALU*

In A, a section of the *CALU* gene model from the Ensembl web pages, with arrows showing the homologous exclusively spliced exons for variants 001 and 002. In fact there are three splice events shown in this image, the homologous substitution, an N-terminal extension, which comes from the extra exon at the 5' end in variants 003 and 005, and which cannot be detected by proteomics data, and a C-terminal substitution that results from skipping the penultimate coding exon in 006 (a C-terminal substitution in protein terms since the frame of the last exon is changed by the exon skip event. In B part of the pairwise alignment between the protein sequences of variants 001 (ENST00000249364) and 002 (ENST00000449187) showing the similarity between the two protein sequences that results from the homologous splicing event. Homologous exon events can also occur with the 5' and 3' exons.
