# Supplementary material for: Alternatively Spliced Homologous Exons Have Ancient Origins and Are Highly Expressed at the Protein Level
Source: PLoS Comput Biol. 2015 Jun 10;11(6):e1004325. doi: 10.1371/journal.pcbi.1004325 (PMC4465641; doi:10.1371/journal.pcbi.1004325)
Supplement: S17 Fig — A. Seed alignment for Pfam domain PF12424 (Plasma membrane calcium transporter ATPase C terminal). B. Structure resolved for this domain, from PDB structure 2KNE, chain B, which is a single helix, bound to calmodulin. C. The alignment between the two homologous isoforms of ATP2B1 identified in the experiments. The blue and red arrows show where the Pfam seed alignment begins (blue) and where the conserved section of the Pfam domain ends (red). The Pfam domain is highly conserved over the first part of the alignment (the part of the alignment that coincides with the crystalised helix structure), but has little conservation at the C-terminal end. The (distant) homology detected for the ATP2B1 isoforms (and all other members of this family) is clear up to the residue indicated with the red arrow, but it not conserved after and therefore the splice event would break the domain. However, the evidence from the PDB structure (and the lack of conservation in the Pfam seed alignment itself) suggests that the Pfam alignment should not be extended beyond the red arrow. In fact the residues before the first N-terminal residue of PF12424 are just as conserved as the residues between the red and blue arrows, suggesting that perhaps the plasma membrane calcium transporter ATPase C terminal domain should also be extended towards the N-terminal end. (PDF) [file pcbi.1004325.s020.pdf]

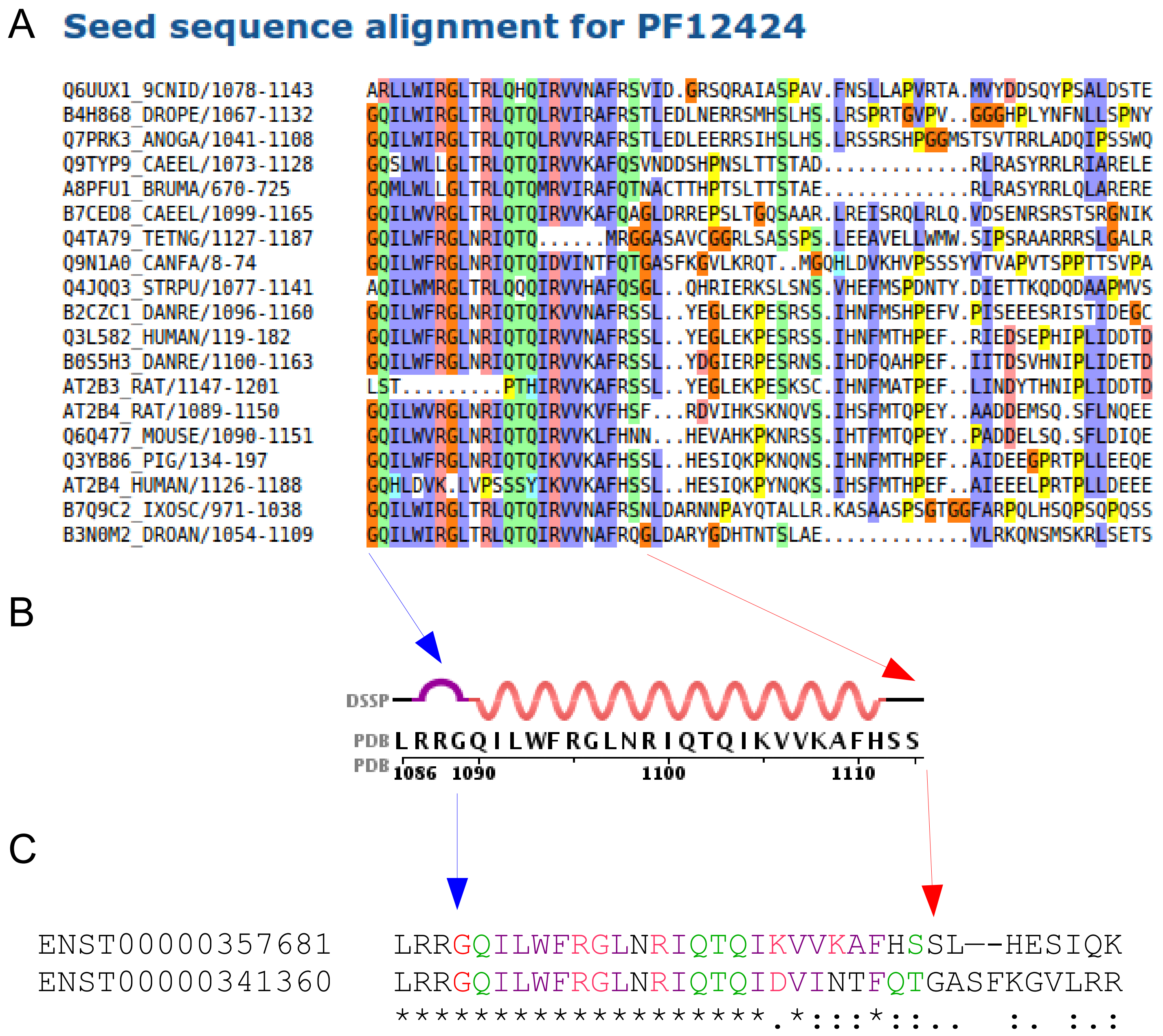
A
Seed sequence alignment for PF12424
Q6UUX1_9CNID/1078-1143 ARLLWIRGLTRLQHQIRVVNAFRSVID.GRSQRAIASPAV.FNSLLAPVRTA.MVYDDSQYPSALDSTE
B4H868_DROPE/1067-1132 GQILWIRGLTRLQTQLRVIRAFRSTLEDLNERRSMHSLHS.LRSPRTGVPV..GGGHPLYNFNLLSPNY
Q7PRK3_ANOGA/1041-1108 GQILWIRGLTRLQTQLRVVRAFRSTLEDLEERRSIHSLHS.LRSSRSHPGGMSTSVTRRLADQIPSSWQ
Q9TYP9_CAEEL/1073-1128 GQSLWLLGLTRLQTQIRVVKAFQSVNDDSHPNSLTTSTAD.............RLRASYRRLRIARELE
A8PFU1_BRUMA/670-725 GQMLWLLGLTRLQTQMRVIRAFQTNACTTHPTSLTTSTAE.............RLRASYRRLQLARERE
B7CED8_CAEEL/1099-1165 GQILWVRGLTRLQTQIRVVKAFQAGLDRREPSLTGQSAAR.LREISRQLRLQ.VDSENRSRSTSRGNIK
Q4TA79_TETNG/1127-1187 GQILWFRGLNRIQTQ......MRGGASAVCGGRLSASSPS.LEEAVELLWMW.SIPSRAARRRSLGALR
Q9N1A0_CANFA/8-74 GQILWFRGLNRIQTQIDVINTFQTGASFKGVLKRQT..MGQHLDVKHVPSSSYVTVAPVTSPPTTSVPA
Q4JQQ3_STRPU/1077-1141 AQILWMRGLTRLQQQIRVVHAFQSGL..QHRIERKSLSNS.VHEFMSPDNTY.DIETTKQDQDAAPMVS
B2CZC1_DANRE/1096-1160 GQILWFRGLNRIQTQIKVVNAFRSSL..YEGLEKPESRSS.IHNFMSHPEFV.PISEEESRISTIDEGC
Q3L582_HUMAN/119-182 GQILWFRGLNRIQTQIRVVNAFRSSL..YEGLEKPESRSS.IHNFMTHPEF..RIEDSEPHIPLIDDTD
B0S5H3_DANRE/1100-1163 GQILWFRGLNRIQTQIRVVKAFRSSL..YDGIERPESRNS.IHDFQAHPEF..IITDSVHNIPLIDETD
AT2B3_RAT/1147-1201 LST.........PTHIRVVKAFRSSL..YEGLEKPESKSC.IHNFMATPEF..LINDYTHNIPLIDDTD
AT2B4_RAT/1089-1150 GQILWVRGLNRIQTQIRVVKVFHSF...RDVIHKSKNQVS.IHSFMTQPEY..AADDEMSQ.SFLNQEE
Q6Q477_MOUSE/1090-1151 GQILWVRGLNRIQTQIRVVKLFHNN...HEVAHKPKNRSS.IHTFMTQPEY..PADDELSQ.SFLDIQE
Q3YB86_PIG/134-197 GQILWFRGLNRIQTQIKVVKAFHSSL..HESIQKPKNQNS.IHNFMTHPEF..AIDEEGPRTPLLEEQE
AT2B4_HUMAN/1126-1188 GQHLDVK.LVPSSSYIKVVKAFHSSL..HESIQKPYNQKS.IHSFMTHPEF..AIEEELPRTPLLDEEE
B7Q9C2_IXOSC/971-1038 GQILWIRGLTRLQTQIRVVNAFRSNLDARNNPAYQTALLR.KASAASPSGTGGFARPQLHSQPSQPQSS
B3N0M2_DROAN/1054-1109 GQILWIRGLTRLQTQIRVVNAFRQGLDARYGDHTNTSLAE.............VLRKQNSMSKRLSETS
B
DSSP
PDB LRRGQILWFRGLNRIQTQIKVVKAFHSS
PDB 1086 1090 1100 1110
C
ENST00000357681 LRRGQILWFRGLNRIQTQIKVVKAFHSSL--HESIQK
ENST00000341360 LRRGQILWFRGLNRIQTQIDVINTFQTGASFKGVLRR
******************.*:::*:::. :. :.:

Figure S17. The Pfam domain broken in the splice variants of *ATP2B1* and *ATP2B4*

A. Seed alignment for Pfam domain PF12424 (Plasma membrane calcium transporter ATPase C terminal). B. Structure resolved for this domain, from PDB structure 2KNE, chain B, which is a single helix, bound to calmodulin. C. The alignment between the two homologous isoforms of *ATP2B1* identified in the experiments. The blue and red arrows show where the Pfam seed alignment begins (blue) and where the conserved section of the Pfam domain ends (red). The Pfam domain is highly conserved over the first part of the alignment (the part of the alignment that coincides with the crystalised helix structure), but has little conservation at the C-terminal end. The (distant) homology detected for the *ATP2B1* isoforms (and all other members of this family) is clear up to the residue indicated with the red arrow, but it not conserved after and therefore the splice event would break the domain. However, the evidence from the PDB structure (and the lack of conservation in the Pfam seed alignment itself) suggests that the Pfam alignment should not be extended beyond the red arrow. In fact the residues before the first N-terminal residue of PF12424 are just as conserved as the residues between the ed and blue arrows, suggesting that perhaps the plasma membrane calcium transporter ATPase C terminal domain should also be extended towards the N-terminal end.
